# Motor-like Tics are Mediated by CB_2_ Cannabinoid Receptor-dependent and Independent Mechanisms Associated with Age and Sex

**DOI:** 10.1007/s12035-022-02884-6

**Published:** 2022-06-06

**Authors:** Victoria Gorberg, Veronika Borisov, Iain R. Greig, Roger G. Pertwee, Peter McCaffery, Sharon Anavi-Goffer

**Affiliations:** 1 School of Medicine, Medical Sciences and Nutrition, Institute of Medical Sciences, University of Aberdeen, Aberdeen, UK; 2 Technion Faculty of Medicine, Technion-Israel Institute of Technology, Haifa, Israel

**Keywords:** Tic disorder, Premonitory urges, Anandamide, GPR55, Tetrahydrocannabivarin (THCV), Cannabidivarin (CBDV), α/β-Hydrolase domain-containing 6 (ABHD6)

## Abstract

Δ^9^-Tetrahydrocannabinol (Δ^9^-THC) inhibits tics in individuals with Tourette syndrome (TS). Δ^9^-THC has similar affinities for CB_1_/CB_2_ cannabinoid receptors. However, the effect of HU-308, a selective CB_2_ receptor agonist, on repetitive behaviors has not been investigated. The effects of 2,5-dimethoxy-4-iodoamphetamine (DOI)-induced motor-like tics and Δ^9^-THC were studied with gene analysis. The effects of HU-308 on head twitch response (HTR), ear scratch response (ESR), and grooming behavior were compared between wildtype and CB_2_ receptor knockout (CB_2_^−/−^) mice, and in the presence/absence of DOI or SR141716A, a CB_1_ receptor antagonist/inverse agonist. The frequency of DOI-induced repetitive behaviors was higher in CB_2_^−/−^ than in wildtype mice. HU-308 increased DOI-induced ESR and grooming behavior in adult CB_2_^−/−^ mice. In juveniles, HU-308 inhibited HTR and ESR in the presence of DOI and SR141716A. HU-308 and beta-caryophyllene significantly increased HTR. In the left prefrontal cortex, DOI increased transcript expression of the CB_2_ receptor and GPR55, but reduced fatty acid amide hydrolase (FAAH) and α/β-hydrolase domain-containing 6 (ABHD6) expression levels. CB_2_ receptors are required to reduce 5-HT_2A/2C_-induced tics in adults. HU-308 has an off-target effect which increases 5-HT_2A/2C_-induced motor-like tics in adult female mice. The increased HTR in juveniles induced by selective CB_2_ receptor agonists suggests that stimulation of the CB_2_ receptor may generate motor tics in children. Sex differences suggest that the CB_2_ receptor may contribute to the prevalence of TS in boys. The 5-HT_2A/2C_-induced reduction in endocannabinoid catabolic enzyme expression level may explain the increased endocannabinoids’ levels in patients with TS.

**Supplementary Information:**

The online version contains supplementary material available at 10.1007/s12035-022-02884-6.

## Introduction

Δ^9^-Tetrahydrocannabinol (Δ^9^-THC) inhibits motor tics in adolescent and adult individuals with Tourette syndrome (TS), with onset around age 6 years and with a 3:1 boy:girl ratio [1–4]. In rodents, Δ^9^-THC dose-dependently reverses motor-like tics (sudden, repetitive twitches or movements that may represent Tourette syndrome motor tics), head twitch response (HTR), ear scratch response (ESR), and grooming behavior, after induction of tic-like behavior with 2,5-dimethoxy-4-iodoamphetamine (DOI), a highly potent agonist of the serotonin 5-HT_2A/2C_ receptors [5, 6]. Δ^9^-THC is a partial agonist of the cannabinoid CB_1_ and CB_2_ receptors, but can also act on other receptors, e.g., GPR55 [7, 8]. While the CB_1_ receptor is highly expressed on the surface of central and peripheral neurons, the cannabinoid CB_2_ receptor is highly expressed on cells of the immune system and activated microglia, but low expression levels of the CB_2_ receptor have been reported in the adult CNS under healthy physiological conditions [9, 10].

Evidence exists for the expression of functional CB_2_ receptors on neurons in different brain regions, including the striatum and brainstem, where it regulates dopamine release, while CB_2_ receptor expression levels in the brain can be significantly upregulated during CNS pathologies [9, 11–16]. For example, in adult male mice, exposure to JWH-133 (10, 20 mg/kg, intraperitoneally (i.p.)), a selective CB_2_ receptor agonist, reduces adult locomotor activity [14]. Similarly, JWH-133 reduces locomotor activity induced by cocaine [17]. In adult male mice, HU-308 (2.5, 5 mg/kg, i.p.), another selective CB_2_ receptor agonist, reduces dyskinesia-like behavior in a model of Parkinson’s disease [13]. However, HU-308 (40 mg/kg, i.p.) has no effect on the locomotor activity of adult Sabra female mice [18].

Thus, it appears that there is a complex mechanism for the control of motor activity by the CB_2_ receptor and sex may contribute to these differences. Different selective CB_2_ receptor agonists (e.g., HU-308, JWH-133, HU-910) have been shown to modulate distinct signaling pathways [19]. Questioning their specificity, CB_2_ receptor agonists can also modulate other targets, including receptors other than the cannabinoid receptors, as well as transporters and enzymes [19]. Despite these considerations, HU-308 was selected as one of the best three selective CB_2_ receptor agonists to study the role of the CB_2_ receptor in diseases [20–23].

Similar to its effects on DOI-induced repetitive behaviors, Δ^9^-THC dose-dependently reduces HTR and ESR after the administration of SR141716A, a selective CB_1_ receptor antagonist/inverse agonist, to juvenile male albino ICR mice [24]. Like DOI, SR141716A administration has been proposed as a model for tic-like behavior, but similar model limitations as described before are applied to the SR141716A-induced repetitive behaviors model system [6]. The administration of SR141716A (rimonabant, Acomplia®, Zimulti®) to humans produces psychiatric and neurologic adverse effects such as suicidality, depressed mood, anxiety, insomnia, stress, and seizures. However, motor tics and premonitory urges were not observed in humans after taking rimonabant. In mice, SR141716A dose-dependently induces motor-like tics and premonitory urge-like behavior, effects which are reversed by the 5-HT_2A/2C_ antagonist SR46349B [25]. However, in contrast to DOI, SR141716A does not increase grooming behavior in juvenile ICR mice [25], though it increases serotonin and dopamine release [26]. However, this appears to be species-dependent, as in rats, SR141716A increases grooming behavior [27].

The CB_2_ receptor makes a significant contribution to the control of locomotor activity [13, 14]. Despite the large body of work pointing to the role of the CB_2_ receptor in different diseases, the effect of CB_2_ selective agonists on stereotypical, repetitive behaviors has not been studied. As CB_2_ receptor expression is developmentally regulated, with the expression level being high after birth and very low in the adult brain [9, 28–30], it was important to study the effects of selective CB_2_ receptor ligands at different ages. The possible contribution of the CB_2_ receptor to the skewed ratio between boys and girls in TS was studied by testing motor-like tics in juvenile males and females.

## Materials Methods

### Animals

All experiments were approved by the Institutional Animal Use and Care Committees of Tel-Aviv University and Ariel University and were in accordance with the UK Home Office, EU directive 63/2010E, and the Animal (Scientific Procedures) Act 1986.

The specificity of HU-308 was tested in CB_2_ receptor knockout (CB_2_^−/−^) mice (JAX #005,786), purchased from Jackson Laboratory, USA, and genotyped according to the instructions provided by the company. The experiments were performed as indicated in > 7.5-week-old (7 males and 7 females, adult) CB_2_^−/−^ mice.

Screening of the effects of HU-308 at different ages was conducted in C57BL/6 J (OlaHsd sub-strain). This strain was used in our previous study to screen the effects of Δ^9^-THC and CBD [6]. C57BL/6 J (OlaHsd sub-strain) male and female mice were purchased from Envigo, Israel or UK. The experiments were performed as indicated in 3-week-old (201 males and 66 females, unweaned, juvenile), 6-week-old (63 males and 28 females, pubertal, young adult), and > 7.5-week-old (11 males and 7 females, adult) mice.

### Drugs

SR141716A was synthesized by IRG, University of Aberdeen (according to US Patent 5,462,960). (R)(-)-DOI hydrochloride (CAS 82864–02-6), DMSO, and Kolliphor® EL were from Sigma-Aldrich (Rehovot, Israel). Ethanol was from Merck, Germany. HU-308 was from Tocris, UK. E-BCP was from Kanata Enterprises, India (99%). Δ^9^-THC (98%) was kindly provided by Prof. Mechoulam (The Hebrew University, Israel). DOI (1 mg/kg) was dissolved in saline. HU-308 (0.2 mg/kg, 1 mg/kg, 5 mg/kg), E-BCP (1 mg/kg, 5 mg/kg, 10 mg/kg), and Δ^9^-THC (5 mg/kg) were dissolved in vehicle made of 0.6:1:1.84 DMSO: Kolliphor® EL:saline. SR141716A (5 mg/kg, 10 mg/kg, 20 mg/kg) was dissolved in vehicles 0.6:1:1.84 ethanol: Kolliphor® EL: saline or DMSO: Kolliphor® EL:saline, as indicated in legends. The drugs were freshly prepared, aliquoted, and stored at − 20 ℃ for up to 3 months. Each aliquot was discarded after one use. Drugs were injected intraperitoneally (i.p.). All injections were made in a volume of 10 µl/g.

### Experimental Procedures for Head Twitch Response (HTR), Ear Scratch Response (ESR), and Grooming Behavior Measurement

The experimental procedures for the DOI model system and for randomization have been previously described [6] and are detailed in the Supplementary Information.

### Open Field Test

The test was performed similarly to the methods previously described [18]. The method is described in the Supplementary Information.

### Marble Burying Test

The test was conducted similarly to methods previously published [31]. The method is described in the Supplementary Information.

### Reverse Transcription and RT-PCR

In juvenile mice, the effects of DOI or 5 mg/kg ∆^9^-THC on genes of the endocannabinoid system were tested. The method is described in the Supplementary Information. The sequences of primers used in this study are provided in Table 1.

**Table 1:** Sequences of primers used for mouse RT-PCR analyses

Target	Forward (F)/reverse (R)	Sequence of primers
GAPDH	F	AACTTTGGCATTGTGGAAGG
	R	ACACATTGGGGGTAGGAACA
CB_1_ receptor	F	TCTTAGACGGCCTTGCAGAT
	R	AGGGACTACCCCTGAAGGAA
CB_2_ receptor	F	GAAACAGCCCGAGTCAGAAG
	R	GAGCCTGCCATTCTTACAGG
GPR55	F	GTCCATATCCCCACCTTCCT
	R	CATCTTGAATGGGAGGGAGA
MAGL	F	CAGAGAGGCCAACCTACTTTTC
	R	ATGCGCCCCAAGGTCATATTT
FAAH	F	GGAAGTGAACAAAGGGACCA
	R	TCCCTGCAGCTTCAGTACCT
ABHD6	F	CCTTGATCCCATCCACCCCGGA
	R	CCCGGACACATCAAGCACCTGG

*GAPDH*, glyceraldehyde 3-phosphate dehydrogenase; *CB*_*1*_* receptor*, cannabinoid CB_1_ receptor; *CB*_*2*_* receptor*, cannabinoid CB_2_ receptor; *GPR55*, G protein-coupled receptor 55; *MAGL*, monoacylglycerol lipase; *FAAH*, fatty acid amide hydrolase; *ABHD6*, α/β-hydrolase domain-containing 6

### Statistical Analysis

All data were expressed as a mean ± SEM. *P* < 0.05 was considered statistically significant. Data were analyzed with GraphPad Prism version 8 (GraphPad, San Diego, CA). Line curves of HTR, ESR, grooming, ambulation, and rearing behaviors were analyzed by two-way analysis of variance (ANOVA), followed by Bonferroni’s post hoc test. Post hoc tests were run only if the *F* ratio was significant, as indicated below (*P < 0.05). The % Frequency of HTR, ESR, and grooming behavior was calculated as previously described [6]. Bar graphs of the number of buried and moved marbles, total distance, duration in the center of the cage, frequency and latency to center, and body weight were analyzed by one-way ANOVA or Student’s *t-*test, as indicated in legends. Bar graphs of gene expression levels were analyzed by Student’s *t*-test, unpaired, followed by Welch’s correction if the low variability within the control group resulted in a significant *F*-test, two-tailed (or one-tailed if the *t*-tests with or without Welch’s correction disagreed).

## Results

### *Effects of HU-308 on DOI-induced Repetitive Behaviors in Adult CB*_*2*_^*−/−*^* Mice*

**Fig. 1:**
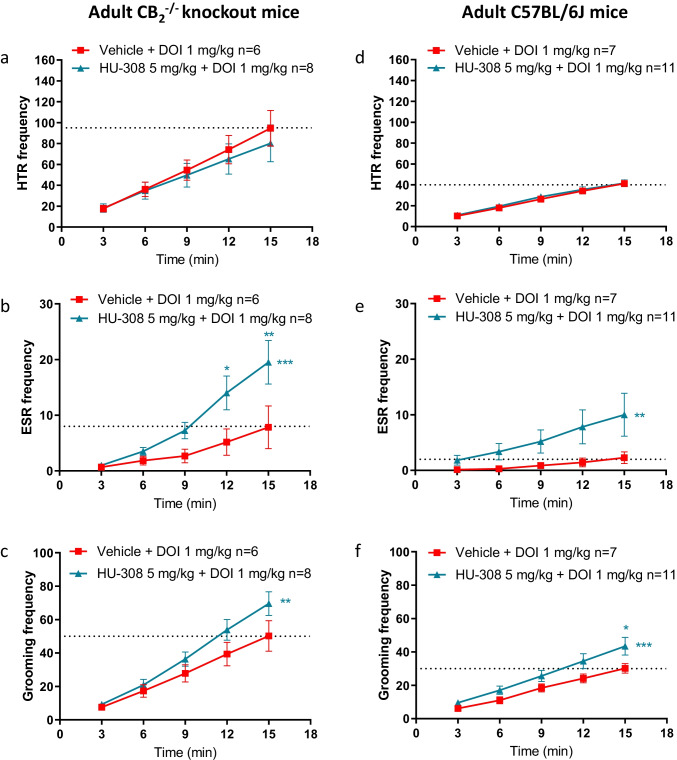
Effects of DOI in the presence or absence of HU-308 (5 mg/kg) on HTR (**a**, **d**), ESR (**b**, **e**), and grooming behavior (**c**, **f**) in adult wildtype (WT) and CB_2_^−/−^ knockout mice (CB_2_^−/−^ mice). In **a**–**c**, the effects of HU-308 on DOI in CB_2_^−/^^−^ mice. In **d**–**f**, the effects of HU-308 on DOI in WT mice. Data represent mean ± SEM. *n* represents the number of animals in each group. The experiment was independently repeated a number of times according to the lowest *n* number. Two-way ANOVA analysis of variance followed by Bonferroni’s test for multiple comparisons was performed by GraphPad Prism 8. Asterisks aside from the graph are *p* value summary vs. vehicle + DOI group. Asterisks along the curve are *p* values of multiple comparisons (at a time point) of each dose vs. vehicle + DOI group. *P < 0.05; **P < 0.01; ***P < 0.001 significantly different

In order to determine the on- versus off-target effects of HU-308 [19], the effects of DOI (1 mg/kg)-induced repetitive behaviors in the presence or absence of HU-308 (5 mg/kg) were tested in CB_2_^−/−^ mice. HU-308 has neuroprotective effects [13, 21, 23], and activation of the CB_2_ receptor inhibits dopamine release [15]; therefore, we expected that HU-308 will reduce the DOI-induced motor-like tics. Surprisingly, the results show that in adult CB_2_^−/−^ mice, HU-308 (5 mg/kg) had no effect on DOI-induced HTR and significantly increased DOI-induced ESR and grooming behavior in adult CB_2_^−/−^ mice (Fig. 1a–c, respectively). These results show that the enhancing effects of HU-308 on DOI-induced repetitive behaviors in adult mice were not CB_2_ receptor-mediated. Sex comparison of the effect of HU-308 in adult CB_2_^−/−^ mice suggests that females were more sensitive than males (Supplementary Figs. S1 and S2).

### Effects of HU-308 on DOI-induced Repetitive Behaviors in Adult Mice

To better understand if these enhancing effects of HU-308 on DOI-induced repetitive behaviors in adult mice were not dependent on the modulation of the CB_2_ receptor, we repeated this experiment in adult mice from another strain. We tested the effects of HU-308 on DOI-induced repetitive behaviors in a sub-strain of wildtype (WT) C57BL/6 J mice, which was used for subsequent experiments in juvenile mice. The results show that in adult WT mice, HU-308 (5 mg/kg) had no effect on DOI-induced HTR but significantly increased DOI-induced ESR and grooming behavior (Fig. 1d–f, respectively), replicating our results in CB_2_^−/−^ mice. The effects of HU-308 on DOI-induced repetitive behaviors in young adult mice are detailed in the Supplementary Information (Supplementary Figs. S3, S4, and S5).

### Effects of HU-308 on DOI-induced Repetitive Behaviors in Juvenile Mice

**Fig. 2:**
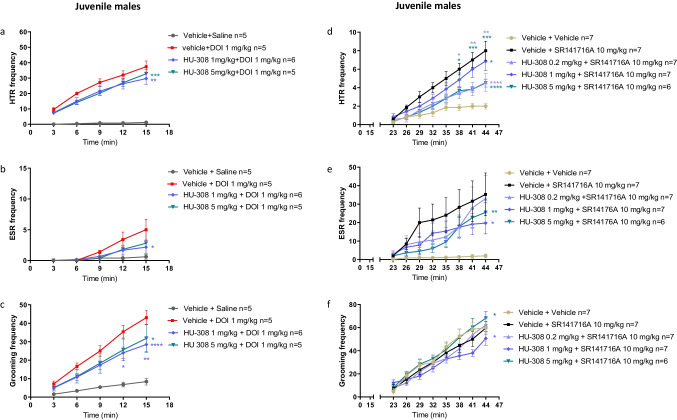
Effects of HU-308 (1 mg/kg, 5 mg/kg) on DOI (1 mg/kg)-induced HTR (**a**), ESR (**b**), and grooming behavior (**c**) in juvenile males. HU-308 (0.2 mg/kg) had no effects (Supplementary Fig. S6). Effects of HU-308 (0.2 mg/kg, 1 mg/kg, 5 mg/kg) on SR141716A (10 mg/kg)-induced HTR (**d**), ESR (**e**), and grooming behavior (**f**) in juvenile males. Data represent mean ± SEM. *n* represents the number of animals in each group. The experiment was independently repeated a number of times according to the lowest *n* number. Two-way ANOVA analysis of variance followed by Bonferroni’s test for multiple comparisons was performed by GraphPad Prism 8. Asterisks aside from the graph are *p* value summary vs. vehicle + DOI group. Asterisks along the curve are *p* values of multiple comparisons (at a time point) of each dose vs. vehicle + DOI or vs. vehicle + SR141716A group. *P < 0.05; **P < 0.01; ***P < 0.001; ****P < 0.0001 significantly different

We expected to find similar results in juvenile mice. Surprisingly, in juvenile male mice, HU-308 (1 mg/kg, 5 mg/kg) reduced DOI-induced HTR, ESR, and grooming behavior (Fig. 2a–c). The DOI-induced HTR was significantly reduced by 21% and 13%, respectively (Fig. 2a, *P* < 0.05). The DOI-induced ESR was reduced by 64% (*P* < 0.05) and 50%, respectively (Fig. 2b). The DOI-induced grooming behavior was significantly reduced by 42% and 32%, respectively (Fig. 2c, *P* < 0.05). Compared with the results in adult mice, these results showed that in juvenile mice, HU-308 inhibits repetitive behaviors.

**Fig. 3:**
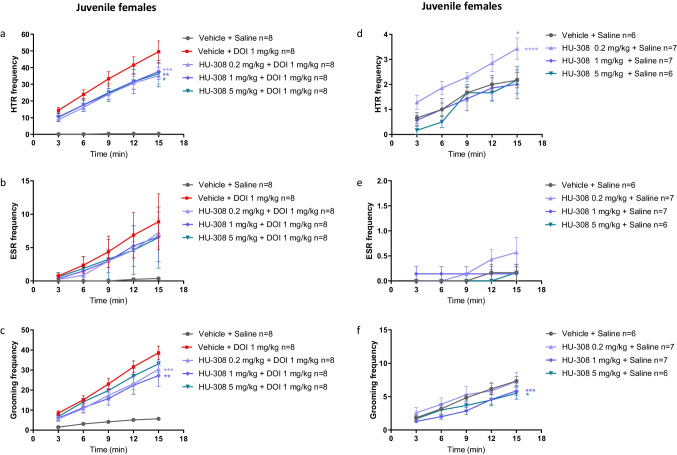
Effects of HU-308 (0.2 mg/kg, 1 mg/kg, 5 mg/kg) on DOI (1 mg/kg)-induced HTR (**a**), ESR (**b**), and grooming behavior (**c**) in juvenile females. Effects of HU-308 alone (0.2 mg/kg, 1 mg/kg, 5 mg/kg) on basal HTR (**d**), ESR (**e**), and grooming behavior (**f**) in juvenile females. Data represent mean ± SEM. *n* represents the number of animals in each group. The experiment was independently repeated a number of times according to the lowest *n* number. Two-way ANOVA analysis of variance followed by Bonferroni’s test for multiple comparisons was performed by GraphPad Prism 8. In **a**–**c**, asterisks aside the graph are *p* value summary vs. vehicle + DOI group. Asterisks along the curve are *p* values of multiple comparisons (at a time point) of each dose vs. vehicle + DOI group. In **d**–**f**, asterisks aside from the graph are *p* value summary vs. control group (vehicle + vehicle). Asterisks along the curve are *p* values of multiple comparisons (at a time point) of each dose vs. the control group. *P < 0.05; **P < 0.01; ***P < 0.001; ****P < 0.0001 significantly different

Age dependency was also demonstrated in female mice. In juvenile females, HU-308 (1 mg/kg, 5 mg/kg) significantly reduced DOI-induced HTR, resulting in 24% and 27% inhibition, respectively (Fig. 3a). HU-308 (0.2 mg/kg, 1 mg/kg, 5 m/kg) had no significant effect on DOI-induced ESR (Fig. 3b), and the effect of HU-308 (1 mg/kg, 5 mg/kg) on DOI-induced grooming behavior resulted in inhibition of 34% (*P* < 0.05) and 17%, respectively (Fig. 3c).

Thus, in contrast to its profound effects to enhance DOI-induced ESR and grooming behavior in adult females, in juveniles, HU-308 (5 mg/kg) significantly inhibited the effects of DOI both in males and females. However, females seemed more sensitive because HU-308 (0.2 mg/kg) had no effect on DOI-induced repetitive behaviors in juvenile males (Supplementary Fig. S6a–c), while it significantly reduced the DOI-induced HTR and grooming behavior in juvenile females (*P* < 0.05; Supplementary Fig. S6d–f), resulting in 29% and 25% inhibition, respectively. Average body weight was not different between groups (Supplementary Fig. S5).

### Effects of HU-308 on SR141716A-induced Repetitive Behaviors in Juveniles

In the presence of SR141716A, HU-308 (0.2 mg/kg, 1 mg/kg, 5 mg/kg) significantly decreased the frequency of HTR (Fig. 2d; *P* < 0.05), resulting in an inhibition of 57%, 19%, and 58%, respectively. In the presence of SR141716A, HU-308 (1 mg/kg, 5 mg/kg) significantly decreased the frequency of ESR (Fig. 2b; *P* < 0.05), resulting in an inhibition of 47%, and 29%, respectively. However, HU-308 (0.2 mg/kg, 1 mg/kg, 5 mg/kg) had no effect on grooming behavior in juvenile male mice (Fig. 2c). Average body weight was not different between groups (Supplementary Fig. S5). The effects of the vehicles (ethanol vs. DMSO) on SR141716A-induced repetitive behaviors are shown in the Supplementary Information (Supplementary Fig. S7a–c). SR141716A, dissolved in ethanol, dose-dependently increased HTR and ESR behaviors but not grooming behavior (Supplementary Fig. S7a–c). These results replicate another study [25].

Collectively, these results show that, in two model systems, HU-308 inhibits repetitive behaviors in juveniles. Therefore, we next studied its effects on basal repetitive behaviors, important to determine because this will impact its potential “therapeutic window.”

### Effect of HU-308 on Basal Repetitive Behaviors in Juvenile Mice

In healthy juvenile females, compared with the basal HTR of the control group, HU-308 alone (0.2 mg/kg) significantly increased HTR (Fig. 3d). HU-308 (1 mg/kg, 5 mg/kg) had no effect on basal HTR (Fig. 3d). HU-308 (0.2 mg/kg, 1 mg/kg, 5 mg/kg) had no effect on basal ESR (Fig. 3e), while HU-308 (1 mg/kg, 5 mg/kg) significantly inhibited basal grooming behavior (Fig. 3f; *P* < 0.05).

**Fig. 4:**
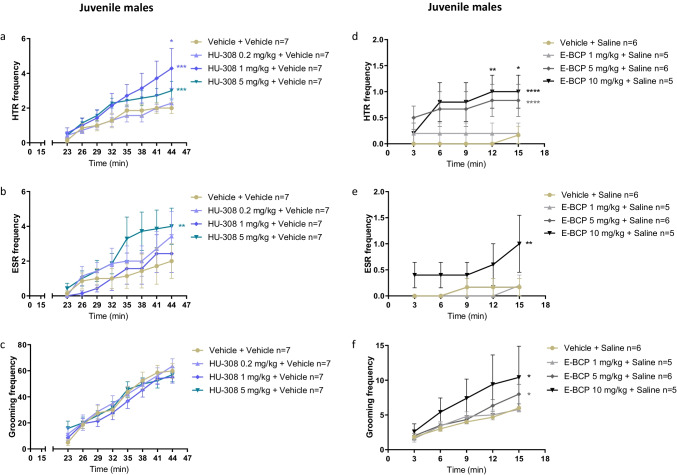
Effects of HU-308 alone (0.2 mg/kg, 1 mg/kg, 5 mg/kg) on basal HTR (**a**), ESR (**b**), and grooming behavior (**c**) in juvenile males. Effects of E-BCP alone (1 mg/kg, 5 mg/kg, 10 mg/kg) on basal HTR (**d**), ESR (**e**), and grooming behavior (**f**) in juvenile males. Data represent mean ± SEM. *n* represents the number of animals in each group. The experiment was independently repeated a number of times according to the lowest *n* number. Two-way ANOVA analysis of variance followed by Bonferroni’s test for multiple comparisons was performed by GraphPad Prism 8. Asterisks aside from the graph are *p* value summary vs. control group (vehicle + vehicle). Asterisks along the curve are *p* values of multiple comparisons (at a time point) of each dose vs. the control group. *P < 0.05; **P < 0.01; ***P < 0.001; ****P < 0.0001 significantly different

In contrast, in healthy juvenile males, HU-308 alone significantly increased the frequency of HTR (Fig. 4a; *P* < 0.05). Compared with the basal HTR of the control group, HU-308 (1 mg/kg, 5 mg/kg) significantly increased HTR, resulting in an increase of 114% and 50% in basal HTR, respectively. Compared with the basal ESR of the control group, HU-308 (5 mg/kg) significantly increased ESR, resulting in an increase of 100% of basal ESR in juvenile male mice (Fig. 4b; *P* < 0.05). Compared with the basal grooming behavior of the control group, HU-308 alone had no effect on basal grooming behavior in juvenile male mice (Fig. 4c). Average body weight was not different between groups (Supplementary Fig. S8c). These results suggest that males are more sensitive than female mice to the effect of selective CB_2_ receptor agonists on basal activity.

We next tested E-BCP, another selective CB_2_ receptor agonist [32]. In healthy juvenile male mice, E-BCP alone (1 mg/kg, 5 mg/kg, 10 mg/kg) dose-dependently increased HTR (Fig. 4d). Compared with basal HTR of the control group, E-BCP alone (5 mg/kg, 10 mg/kg) significantly increased HTR by 400% and 500%, respectively (Fig. 4d; *P* < 0.05). E-BCP alone (10 mg/kg) significantly increased basal ESR by 500% (Fig. 4e). E-BCP alone (5 mg/kg, 10 mg/kg) significantly increased basal grooming behavior by 33% and 73%, respectively (Fig. 4f; *P* < 0.05). The similarity of these results with that of HU-308 on basal repetitive behaviors in juveniles suggests that these effects are indeed CB_2_ receptor-mediated.

In juvenile male mice, HU-308 (1 mg/kg, 5 mg/kg) significantly reduced the number of rears and ambulatory behavior (Supplementary Fig. S10a,b; *P* < 0.05) but not grooming behavior (Supplementary Fig. S10c). Average body weight was not different between groups (Supplementary Fig. S10d). These results are in line with the inhibitory effect of JWH-133 on locomotor activity [14]. In contrast, DOI (1 mg/kg) significantly increased ambulation and rearing behaviors in juveniles (*P* < 0.05; *n* = 6; VG results, not shown) and SR141716A at a dose of 10 mg/kg, but not at a lower dose, increases ambulation behavior and travel distance in adolescent male rodents [33, 34].

Collectively, these results show that HU-308 reduces locomotor activity but significantly increases repetitive behaviors, inducing a phenotype of motor-like tics without hyperactivity in juvenile males, while DOI and SR141716A induce a phenotype of motor-like tics with hyperactivity in juvenile males.

### *DOI and ∆*.^*9*^*-THC Induce Left Lateralization in the Endocannabinoid System*

**Fig. 5:**
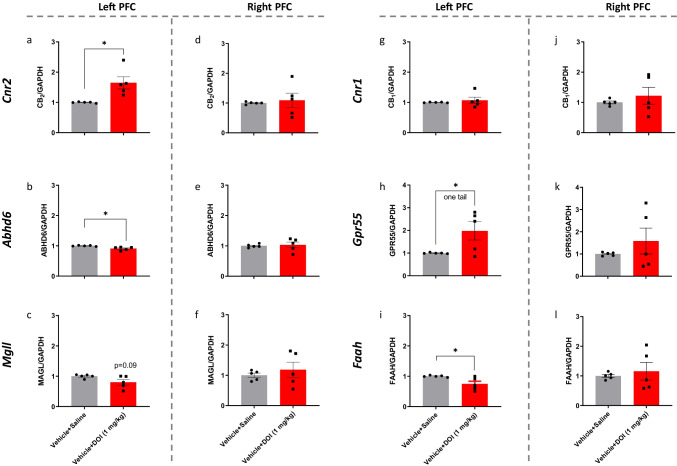
Effects of DOI (1 mg/kg) on the mRNA expression level of elements of the endocannabinoid system and GPR55 in the left (**a**–**c**, **g**–**i**) and right (**d**–**f**, **j**–**l**) prefrontal cortex of juvenile male mice. The experiment was independently repeated 5 times. Expression level was normalized to GAPDH and expressed relative to the control group (vehicle + saline). Expression level was compared with this of the control group (vehicle + saline) and analyzed with Student’s *t-*test, unpaired, two tails (or one tail as indicated), followed by Welch’s correction. *P < 0.05 significantly different

Further support for the involvement of the CB_2_ receptor in juvenile males comes from an RT-PCR study, which focused on the dorsolateral prefrontal cortex (PFC), because in a Genome-Wide Association Study (GWAS), significant genetic mutations in patients with TS found in the PFC and have raised interest in this region [35]. In our study, the CB_2_ receptor expression level was significantly increased by DOI in the left but not in the right PFC (Fig. 5a, d *P* < 0.05).

DOI significantly altered the mRNA expression level of elements of the endocannabinoid system in the left but not in the right PFC (Fig. 5). In addition to the increased expression level of the *Cnr2* gene (encoding the CB_2_ receptor), the expression level of *Gpr55* (encoding gene of GPR55) was significantly increased by DOI in the left, but not in the right, PFC (Fig. 5h, k). However, *Cnr1* (encoding gene of CB_1_ receptor) expression levels were not affected by DOI (Fig. 5g, j). In line with these results, genetic variations of the *CNR1* gene in patients were not correlated with TS [36], further supporting that DOI-induced motor-like tics may closely model TS.

In contrast, *Abhd6* (encoding gene of ABHD6) and *Faah* (encoding gene of FAAH) expression levels were significantly decreased by DOI in the left, but not in the right, PFC (Fig. 5b, i vs. Fig. 5e, l). In the left PFC, DOI reduced the expression level of *Mgll* (encoding gene of MAGL) (*P* = 0.09; Fig. 5c, f).

**Fig. 6:**
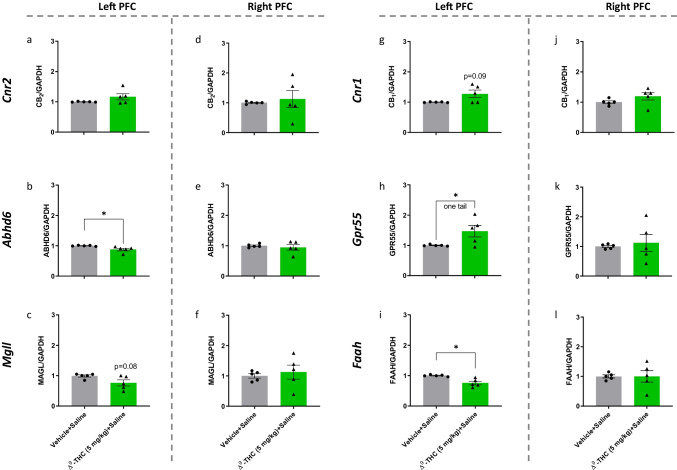
Effects of ∆.^9^-THC (5 mg/kg) on the mRNA expression level of elements of the endocannabinoid system and GPR55 in the left (**a**–**c**, **g**–**i**) and right (**d**–**f**, **j**–**l**) prefrontal cortex of juvenile male mice. The experiment was independently repeated 5 times. Expression level was normalized to GAPDH and expressed relative to the control group (vehicle + saline). Expression level was compared with this of the control group (vehicle + saline) and analyzed with Student’s *t-*test, unpaired, two tails (or one tail as indicated), followed by Welch’s correction. *P < 0.05 significantly different

Similar effects to those of DOI on gene expression were found with ∆^9^-THC alone. This may explain why (1) ∆^9^-THC induces psychosis, similarly to DOI, apart from the effect on CB_2_ receptor expression (Fig. 6a–i), and (2) treatment with ∆^9^-THC only temporarily alleviates the symptoms of TS.

## Discussion

This study demonstrates that the CB_2_ receptor has a role in the control of repetitive behaviors. In support of the contribution of CB_2_ receptors to the control of motor movements are previous studies showing that (1) in rodents, the CB_2_ receptor is expressed on the soma and nerve terminals of dopaminergic neurons projecting from the substantia nigra to the striatum in the nigrostriatal pathway [12, 37, 38] and from the ventral tegmental area (VTA) to the nucleus accumbens in the mesocortical pathway [15]; (2) the CB_2_ receptor controls the release of dopamine in the dorsal striatum (caudate nucleus and putamen) and nucleus accumbens [12, 15]; (3) in healthy animals, the CB_2_ receptor mediates M_4_ muscarinic acetylcholine receptor-induced inhibition of dopamine release [11]; (4) in non-human primates, the CB_2_ receptor is expressed on globus pallidus (internal and external) output neurons of the basal ganglia [39]; (5) in humans, the CB_2_ receptor is expressed by dopaminergic neurons of the substantia nigra pars compacta (SNc) [40], Purkinje neurons as well as neurons of the dentate nucleus, and in the white matter of the cerebellum in patients with loss of motor coordination [41]. Most of these studies have focused on neuronal cells; however, in some of these studies, CB_2_ receptors have been localized on glial cells as well [15, 37, 38, 41], suggesting that the CB_2_ receptor is expressed by neuronal and glial cells in brain areas that control motor function.

In this study, several limitations in the models employed need to be taken into account: (1) DOI and SR141716A are administered systemically, thus affecting multiple brain regions including those that do not cause tics [42–44]; (2) systemic administration of DOI or rimonabant to humans does not lead to the appearance of tics; (3) the tested drugs are used as pre-treatments prior to the administration of DOI or SR141716A. This is not the case in humans, who are treated only after the appearance of symptoms; (4) CB_2_ expression in the CNS changes in pathological diseases but our models use only healthy mice; (5) Tourette syndrome consists of both motor and vocal tics and while DOI induces motor-like tics it does not induce vocal tics [6]. Similarly, administration of SR141716A to juvenile and adult mice does not induce vocalizations; (6) in mice, under the experimental conditions employed, SR141716A does not induce peripheral motor-like tics, making it only a partial model for motor-like tics.

### *A Role for CB*_*2*_* Receptor in Movement Disorders*

Following activation of 5-HT_2A/2C_ receptors by DOI, repetitive behaviors were higher in adult CB_2_^−/−^ than in wildtype mice, and the deletion of CB_2_ receptor reveals its contribution to 5-HT_2A/2C_ receptor-induced repetitive behaviors. Interestingly, CB_2_^−/−^ mice with deleted CB_2_ receptor on dopamine neurons show increased hyperactivity [45]. Previous studies showed that in healthy animals CB_2_ receptor inhibits the release of dopamine [11, 12, 15]. Collectively these results suggest that (1) during healthy brain development, Gαi protein-coupled CB_2_ receptors are required to reduce the magnitude of dopamine release, including when stimulated by activation of 5-HT_2A/2C_ receptors, and (2) this mechanism, in turn, reduces the frequency of repetitive behaviors in healthy animals.

Our results further suggest that losing expression of functional brain CB_2_ receptors will contribute to a robust motor tic phenotype. These results imply that a sudden and profound drop in the cerebral expression level of Gαi protein-coupled CB_2_ receptor during adulthood may possibly contribute to the appearance of adult-onset tic disorders [46]. Vice versa, the severity of motor tics gradually declines through adolescence, and by adulthood, most patients experience a significant reduction in the number of tics [1]. One possible explanation for this is that the cerebral expression level of the Gαi protein-coupled CB_2_ receptor is gradually re-stabilized in adult TS patients with reduced tics.

### HU-308 Increases DOI-induced Motor-like Tics but has a Novel Target in Adult Mice

In the presence or absence of CB_2_ receptor expression, HU-308 significantly increased DOI-induced ESR and grooming behavior, implying that another target mediates the effect of HU-308 on motor-like tics in adult mice. Indeed, HU-308 has a number of off-target receptors including 5-HT_2A_, cholecystokinin 1 (CCK-1), tachykinin 2 (NK2), and angiotensin 1 (AT_1_) receptors, and the dopamine and norepinephrine transporters [19]. Identification of the off-target receptor(s)/transporter(s) of HU-308 in this model may lead to the discovery of a new pathway that regulates motor tics.

### *HU-308 Increase of Motor-like Tics in Juveniles is CB*_*2*_* Receptor-mediated*

Surprisingly, in juveniles, HU-308 alone significantly increased basal repetitive behaviors. The stimulatory effects of HU-308 on basal motor-like tics were mimicked by E-BCP, another CB_2_ receptor-selective agonist. These results suggest a possible role for stimulation of the CB_2_ receptor in the development of motor tics in children. Thus, it is possible that in juveniles, in the presence of basal activity of D_2_ autoreceptors (i.e., lack of dopamine), the CB_2_ receptor will favor the coupling to Gαs protein[12] (extended in the Supplementary Information). This may mean that selective CB_2_ receptor agonists, such as HU-308 and E-BCP, and endogenous CB_2_ receptor agonists, such as 2-arachidonoylglycerol (2-AG), will possibly enhance the release of dopamine in children, resulting in increased frequency of motor tics.

### Behavioral Response to HU-308 is Dependent on Age and Sex

In contrast to the enhancing effect by HU-308 of DOI-induced motor-like tics in adult mice, in juvenile mice, HU-308 inhibited DOI-induced HTR, ESR, and grooming behavior. These inhibitory effects of HU-308 were mimicked in another model system of SR141716A-induced motor-like tics, where HU-308 inhibited SR141716A-induced HTR and ESR. These results suggest that the effect of selective CB_2_ receptor agonists on motor-like tics and urge-like responses is dependent on age. The implications for drug development are that selective CB_2_ receptor ligands should be tested at different developmental stages within the same model.

Our study found that in juvenile mice, (1) HU-308 and E-BCP, selective CB_2_ receptor agonists, enhanced basal repetitive behaviors in juvenile males, suggesting these effects were CB_2_ mediated; (2) the intensity of the effects of HU-308 in females was lower than in males, e.g., HU-308 had a lower or no effect on HTR in juvenile females; (3) HU-308 significantly decreased basal grooming behavior in juvenile females but not in males, suggesting that CB_2_ receptor stimulation may possibly reduce the frequency of caudally located motor tics in juvenile females; (4) HU-308 (0.2 mg/kg) significantly inhibited DOI-induced HTR and grooming behavior in females but not in males.

Collectively, these results suggest that the CB_2_ receptor contributes to the skewed ratio between juvenile males and females with TS, reducing the prevalence of TS in juvenile females. Possible explanations for these results are related to common pathways between sex hormones and cannabinoids [47]. In specific brain areas, estrogen modulates the inhibitory effect of cannabinoids on GABAergic and glutamatergic transmission [48]. In addition, 17-beta-oestradiol increases the CB_2_ receptor expression on osteoclast [49]. Thus, it may be possible that in juvenile females, estrogen modulates GABA release while increased estradiol level may contribute to the increased expression level of the CB_2_ receptor, which in turn may reduce the release of dopamine [14] in the basal ganglia. Revealing the mechanism may explain why juvenile females are, relatively to males, more protected from the generation of motor tics.

### *Activation of 5-HT*_*2A/2C*_* Receptors Induces Lateralization in the Endocannabinoid System*

Activation of 5-HT2_A/2C_ receptors reduced the expression level of transcripts encoding ABHD6 and MAGL enzymes, which hydrolyze the endocannabinoid 2-AG, and FAAH which hydrolyses anandamide. As there can be differences between gene and protein expression, we discuss below the different possible scenarios. In the first scenario, gene and protein expressions are in opposite directions. RNA-binding proteins that regulate translational processes are crucial for proper neuronal function though the control of post-transcriptional events [50]. In our model system, this may result in no change in the protein expression level of the above enzymes or may lead to an actual increase in the expression level of these enzymes, independent of a change of gene transcript. Such an increased enzymatic activity will reduce the level of the above endocannabinoids, damaging neuronal and glia functioning. According to this scenario, small molecules that inhibit these enzymes may lead to the development of new therapeutics for the treatment of motor tics. Such a candidate is ABX-1431, which inhibits MAGL; however, a clinical trial with ABX-1431 in adult patients with Tourette syndrome did not show significant results [51].

In the second scenario, gene and protein expressions are in the same direction. This may result in an increase in 2-AG and anandamide levels. These results suggest the existence of a mechanism for a “sustained” increase of 2-AG and anandamide levels in TS. This is important as a clinical study found increased 2-AG and anandamide levels in the CSF of patients with TS [52]. Another mechanism has been proposed for “acute” increase of 2-AG level, where activation of M_4_ muscarinic acetylcholine receptors expressed on a population of striatal D_1_-expressing medium spiny neurons (MSNs) increases the synthesis of 2-AG, which is then retrogradely released to stimulate presynaptic CB_2_ receptors on dopaminergic terminals [11]. Indeed, activation of 5-HT_2A/2C_ receptors by DOI induces the release of acetylcholine in the prefrontal cortex [53]. Therefore, it is possible that both mechanisms exist in the prefrontal cortex leading to increased 2-AG level. However, while the “acute” mechanism has been associated with the initial response to stress, a fight-or-flight survival mechanism, the “sustained” mechanism has been associated with long-term effects of stress, leading, for example, to memory impairment [54].

Our results imply that this increased 2-AG level may possibly be a result of 5-HT_2A/2C_ receptor stimulation and can start as early as childhood, leading to left prefrontal cortex lateralization in the expression levels of components of the endocannabinoid system. Interestingly, the left dorsolateral prefrontal cortex controls error-related processes, while the left dorsolateral premotor cortex controls accurate movement timing of either hand [55, 56]. Indeed, lateralization in single-hand finger movements, with longer touch duration, shorter movement time, and more errors, has been presented by children with TS and can persist into adulthood [57, 58]. Correlating 2-AG levels in the brain with those of the CSF levels from treated animals and from patients with errors in sequential finger tasks may help to diagnose patients with TS.

### GPR55 Inhibitors as Novel Drugs for TS

Our results suggest that activation of 5-HT_2A/2C_ receptors will increase the expression of both CB_2_ receptor and GPR55. Interestingly, 2-AG is more potent at GPR55 than at CB_1_ and CB_2_ receptors but has a similar efficacy at these receptors [59]. In the periphery, CB_2_ receptors heterodimerize with GPR55 to inhibit GPR55 activity [60]. This suggests that an increase in the number of both receptors may increase the number of heterodimers to reduce GPR55 activity, which in turn may impair movement coordination [61]. The potential increased GPR55 expression supports the development of GPR55 inhibitors to treat TS and suggests that a drug combination of ∆^9^-THC with potent GPR55 inhibitors such as tetrahydrocannabivarin (THCV) and cannabidivarin (CBDV) [7, 31, 62] may provide a more efficacious combination of cannabinoids to treat motor tics and to improve motor coordination in patients with TS.

In another system, similar opposing effects of the CB_1_ receptor (as a tumor suppressor) to GPR55 (as an oncogene) have been documented, in which DNA methylation of the *CNR1* and *GPR55* genes were also differentially regulated in samples from patients with colorectal cancer compared to control samples [63]. Further application of bioinformatics will be important to direct future studies in the field of Tourette syndrome.

## Summary

This study discovered that (1) the deletion of CB_2_ receptor expression enhances repetitive behaviors in adult mice; (2) HU-308 modulates a novel target that increases 5-HT_2A/2C_ receptor-induced repetitive behaviors in adult mice; and (3) stimulation of the CB_2_ receptor by selective agonists enhances repetitive behaviors in juvenile mice. This study suggests that stimulation of the CB_2_ receptor in children may contribute to the appearance of motor tics and to the prevalence of motor tics in boys. The results support the development of CB_2_ receptor and GPR55 inhibitors (i.e., antagonists, inverse-agonists, negative allosteric modulators), but also suggest that development of enzyme enhancers (enzyme potentiators) of ABHD6, MAGL, FAAH enzymes, and possibly their combination with or without a CB_2_ receptor inhibitor and a GPR55 inhibitor will provide alternative approaches to treat patients with TS that are diagnosed with increased 2-AG level.

## Supplementary Information

Below is the link to the electronic supplementary material.

Supplementary file1 (PDF 1.05 MB)

## Data Availability

The data that support the findings of this study are available from the corresponding author upon reasonable request. Some data may not be made available because of privacy or ethical restrictions.

## Abbreviations

∆^9^-THC: ∆^9^-Tetrahydrocannabinol
CBD: Cannabidiol
THCV: ∆^9^-Tetrahydrocannabivarin
CBDV: Cannabidivarin
2-AG: 2-Arachidonoylglycerol
DOI: 2,5-Dimethoxy-4-iodoamphetamine
HTR: Head twitch response
ESR: Ear scratch response
OCD: Obsessive-compulsive disorder
CNS: Central nervous system
PNS: Peripheral nervous system
VTA: Ventral tegmental area
ΔΔCt: Delta-delta Ct
MAGL: Monoacylglycerol lipase
FAAH: Fatty acid amide hydrolase
ABHD6: α/β-Hydrolase domain-containing 6
GPR55: G protein-coupled receptor 55
MSNs: Medium spiny neurons
GWAS: Genome-Wide Association Study
