# Supplementary material for: Motor-like Tics are Mediated by CB2 Cannabinoid Receptor-dependent and Independent Mechanisms Associated with Age and Sex
Source: Mol Neurobiol. 2022 Jun 6;59(8):5070–83. doi: 10.1007/s12035-022-02884-6 (PMC9363400; doi:10.1007/s12035-022-02884-6)
Supplement: Supplementary file 1 — Supplementary file1 (PDF 1.05 MB) [file 12035_2022_2884_MOESM1_ESM.pdf]

Motor-like tics are mediated by CB_2 cannabinoid receptor-dependent and independent mechanisms associated with age and sex

Victoria Gorberg[1], Veronika Borisov[2], Iain R. Greig[1], Roger G. Pertwee[1], Peter McCaffery[1], Sharon Anavi-Goffer[1*]

[1]School of Medicine, Medical Sciences and Nutrition, Institute of Medical Sciences, University of Aberdeen, Aberdeen, UK;

[2] Technion Faculty of Medicine, Technion-Israel Institute of Technology, Haifa, Israel

Running title: Pharmacological activities of HU-308 in mouse models of motor tics

Main manuscript and Supplementary material

*Author for correspondence:

Dr Sharon Anavi-Goffer

Institute of Medical Sciences

University of Aberdeen

UK

AB25 2ZD

E-mail: sharon.anavi-goffer@abdn.ac.uk

Supplementary Methods

Animal housing

All mice, transferred in unequal numbers, were included in the experiments unless their development was atypical (e.g. eyes did not open, abnormalities of the tail or ears, wounds etc.). The mice were housed 6-8/cage in a temperature-controlled room (22-24ºC) on a 12h light-dark cycle. Water and food were available ad libitum. Housing was designed such that for each set of experiments (dose range) mice were housed in the same cage. Matching cage environment reduced variation, enabling a reduction in the number of animals for each study. This housing design habituated the mice to the same environment for 10 days before the experimental day, reducing the variability. Body weights were taken before drug injection on the experimental day. The number of mice in each group, as described in Fig. legends, kept balanced apart from Fig. S1J,K,L where the groups were not well balanced but helped to validate that the results are not significant.

Experimental procedures for head twitch response (HTR), ear scratch response (ESR) and grooming behavior measurement

The experimental procedures for the DOI model system and for randomization have been previously described [1]. Mice (4-8) were habituated in their home cage to the experimental environment for 30-60 min. The dose of DOI was selected after preliminary dose response experiments. Briefly, HU-308 (0.2, 5, 10 mg/kg) was administered 60 min before DOI (1 mg/kg) or saline. Five minutes after placing a mouse into the cage center, counting started for 15 min in three minutes intervals.

SR141716A was dissolved in ethanol to compare its effects with a previous study in juvenile ICR mice [2]. The effects of ethanol and DMSO vehicles on SR141716A-induced

repetitive behaviors in C57BL/6J mice were tested. Mice were randomly injected (i.p.) with SR141716A (5, 10, 20 mg/kg), while control mice were injected with the respective vehicle mixture. Each mouse was immediately placed in the middle of a clear glass cage 30 x 30 x 30 cm. Five minutes after placing a mouse into the cage center, counting started for 39 min in three minute intervals.

To test the effects of HU-308 on repetitive behaviors, each set of experiments included appropriate controls with varying concentrations of HU-308. Each mouse was injected (i.p.) with HU-308 or vehicle (as described in each Fig. legend). After 60 min, a second injection (i.p.) of SR141716A (10 mg/kg) or its respective vehicle (made with ethanol or DMSO as indicated below) was administered. Compared with ICR mice [2], SR141716A affected C57BL/6J mice at a slower rate. In our study, the effect of SR141716A on repetitive behaviors in C57BL/6J mice was more pronounced about 20 min after its injection. Therefore, 20 min after placing a mouse into the cage center, counting was started for an additional 24 min in three minute intervals (total time was 44 min).

The HTR/ESR/grooming behaviors were manually counted by an observer. In all experiments, the number of HTR, ESR and grooming behaviors were counted in the same mouse. In both model systems, HTR was counted every time the mouse had a head twitch, as described previously [1,3]. Shakes and other voluntary head movements were not counted.

ESR was counted each time the mouse scratched itself with its hind limbs, similar to methods previously described [3]. Depending on the frequency of action, there are different methods of counting ESR. In the DOI model system, a new ESR action was added to the total counts only if the mouse moved with all four paws since the previous action. Compared with DOI, SR141716A induced a higher ESR frequency. Therefore, in the SR141716A model system, a new ESR action was added to the total counts on every ESR action, similar to that described previously [2].

Self-grooming was counted each time the mouse groomed any body part with its forelimbs or hind limbs and/or licked and cleaned its tail or nails. However, in C57BL/6J mice, DOI induced rapid and brief grooming actions, while SR141716A induced mainly lengthy and long grooming actions. Therefore, in the DOI model system, a new grooming action was added to the total counts only if the mouse moved with all four paws since the previous action. In contrast, in the SR141716A model system, a new grooming action was added to the total counts when a mouse changed to groom another body part since the previous action. All areas around the head were considered as one part, such that if a mouse started to groom, for example, the ears, and then moved to groom the cheeks, this was counted as one action.

We introduced steps to minimize bias when automatic/full blinding procedures could not be applied. We called these steps ‘a semi-blinding protocol’ which also means the experimental person was semi-blind to the study: (1) Each mouse was randomly injected (i.p.) with the tested drug or with vehicle or with saline before the experiment started; (2) Doses were randomized in each set of experiments (according to Fig. legends); (3) Mice were tested in a random order, which reflects a random order of tested doses; (4) After injecting all the mice in the injection room and leaving them in their cage, the person tested the mice according to the order marked on their tails in the experimental room; (5) HU-308 was tested in different models in the same week; (6) Analysis was performed at the end of the entire experiment.

Open field test

The test was performed similarly to the methods previously described [4]. Mice (8) were habituated in their home cage to the experimental environment for 60 min. After injection of HU-308, each mouse was restored back to its home cage. After 60 min, the mouse was injected a second time with vehicle. Immediately after the second injection the mouse was placed at the center of the experimental cage made of a clear glass 30 X 30 X 30 cm and divided into 4 X 4

identical squares by black marks. The ambulation/rearing/grooming behaviors were manually counted by an observer. Ambulation was counted each time the mouse crossed a square line with its four paws. Rearing was counted each time the mouse reared on its hindlimbs and stretched upward with its front paws (on walls or in air) but not if it groomed itself (no stretching motion). Grooming was counted each time the mouse groomed itself with its front paws at face, tail, nails or other body part. Total grooming was counted for all 20 minutes with no intervals. Ambulation and rearing were counted for 20 min in two minute intervals.

Marble burying test

The marble burying test (MBT) has been suggested as a model for screening potential drugs for anxiety and obsessive-compulsive behavior and is sensitive to Δ^9-THC and high doses of CBD [5,6]. The test was conducted similarly to methods previously published [5]. Mice (6-8 mice) were habituated to the experimental environment for 60 min. After injection of HU-308, each mouse was transferred to a transition cage (1). After 60 min, each mouse was exposed to the experimental cage, a white polycarbonate cage 33 X 27 X 16 cm covered with 5 cm of sawdust (clean + dirty from home cage), for 5 min, and then translocated for 5 min into a transition cage (2) while the marbles were arranged in the experiment cage. Twenty opaque black marbles (14 mm diameter) were evenly spaced in the cage at 4 X 5, at 1 cm from the cage walls, on the top of the sawdust. Using video camera and EthoVision XT 11.5, the cage was photographed before and after the experiment. After this procedure, each mouse was placed at the top left corner and recorded for 30 min.

The MBT was electronically recorded by EthoVision XT 11.5 software (Noldus Information Technology, The Netherlands), using build-in parameters, i.e. no special codes. Using this software, the cage was divided into 9 (3 x 3) equally sized areas, the middle area was considered as the center of the cage for all the parameters of the experiment. The number

of buried and moved marbles was manually analyzed from the pair of photos (before and after the experiment). A marble was considered buried if two-thirds of it was covered with sawdust. The total distance, duration in the center of the cage, frequency and latency to center, which are related to changes in locomotor activity, were electronically quantified by EthoVision XT 11.5 software which produced raw data outputs.

Reverse transcription and RT-PCR

In juvenile mice, the effects of DOI or 5 mg/kg Δ^9-THC on genes of the endocannabinoid system were tested. Two days after each experiment [1], mouse brains were removed, washed and dissected into specific regions in ice-cold HEPES buffer [7]. Tissues were stored in RNAlater reagent (Ambion, U.S.) overnight at 4°C and transferred to −80°C for indefinite storage. Total RNA was isolated from the prefrontal cortex, which is enriched with 5-HT_{2A} receptors, using Tri Reagent (Sigma-Aldrich, Israel). Residual genomic DNA was removed from RNA samples using a TURBO DNA-free Kit according to the manufacturer instruction (Ambion, U.S.). Quantity and quality of the RNA were measured at 260/280 and 260/230 nm absorbance ratio of >2.0 with NanoDrop 2000 (Thermo Scientific, U.S.). Total purified RNA (1 μg) was reversed transcribed into complementary DNA (cDNA) using a high-capacity cDNA kit (Applied Biosystems, U.S.). Comparative Real-Time PCR analysis was performed using a Stratagene Mx3000p real-time PCR system (Agilent, U.S.). Each sample contained 1μl cDNA, 300 nM primers and Power SYBR® Green PCR Master Mix (Applied Biosystems). The cycling parameters were: 10 min incubation phase at 95°C, 40 cycles at 95°C for 15 seconds, annealing at 60°C for 1 min and dissociation at 72°C for 6 seconds with a curve to examine non-specific amplification. Efficiency and specificity of the primers were examined by analysis of cDNA and primer dilution curves and by no-template controls. Gene-specific sequence oligonucleotide primers for: glyceraldehyde-3-phosphate dehydrogenase (GAPDH),

monoacylglycerol lipase (MAGL), fatty acid amide hydrolase (FAAH), α/β-hydrolase domain containing 6 (ABHD6), CB_1 receptor, CB_2 receptor, GPR55, were designed by Primer3, verified by Blast (NCBI) and purchased from Sigma-Aldrich (Israel). The sequences of primers used in this study are provided in Table 1. A total of five experimental sets were examined for which the behavioral results were previously published [1]. Each set of experiments (i.e. experimental groups) was tested on the same plate. Each sample (i.e. animal) was tested in duplicate. Averaged gene expression level was normalized to GAPDH as its mRNA expression level was highly stable between different treatments (ΔCt). The variability between the control animals was calculated from the normalized Ct (ΔCt) values of the control group. Delta-delta Ct (ΔΔCt) was calculated relative to the normalized Ct of control group (Vehicle + Saline). Relative quantitation analysis of gene expression levels was performed using the delta-delta Ct ($2^{-\Delta\Delta Ct}$) method [8], such that all values were relative to the control group which was set as a delta-delta Ct ($2^{-\Delta\Delta Ct}$) of one.

Supplementary results

Effects of HU-308 on DOI-induced repetitive behaviors in adult mice- sex differences

Both sexes contributed to the increase in DOI-induced repetitive behaviors in $CB_2^{-/-}$ mice (Fig. S1). The exploratory results show sex differences for the effects of DOI on repetitive behaviors in $CB_2^{-/-}$ mice, i.e. DOI induced lower frequency of repetitive behaviors in females (Fig. S1a-c) than in males (Fig. S1d-f). In addition, exploratory results showed no sex differences for ESR: HU-308 increased ESR in $CB_2^{-/-}$ (Fig. S1b,e) and WT (Fig. S1h,k) female and male mice, and no sex difference were apparent for HTR in WT mice (Fig. S1g,j). In contrast, exploratory results showed sex differences for the effects of HU-308 on DOI-induced HTR and grooming behavior in $CB_2^{-/-}$ mice (Fig. S1). While HU-308 increased DOI-induced grooming behavior and HTR in females (Fig. S1a,c), it inhibited these behaviors in males (Fig. S1d,f). Body weights were not different between groups (Fig. S2a,b). These results suggest that in adult mice the lack of functional CB_2 receptor signaling makes sex differences in the response to DOI and HU-308 more noticeable.

It is noteworthy that this is not a traditional comparison of basal behavior between knockout and their corresponding WT mice. Yet, we noticed two main differences between the two sub-strains: (1) in adult $CB_2^{-/-}$ mice, DOI-induced repetitive behaviors HTR, ESR and grooming behavior were, respectively, 2.3, 3.4 and 1.7 times significantly higher compared with WT adult mice (Fig. 1a-f; $P < 0.05$, 2-way ANOVA); (2) compared with adult WT mice, the DOI-induced HTR resulted in a vigorous and robust HTR in the $CB_2^{-/-}$ mice.

Effects of HU-308 on DOI-induced repetitive behaviors in young adult mice

We expected to find similar results in young adult mice. However, HU-308 (0.2, 1, 5 mg/kg) had no effect on DOI-induced HTR and grooming behavior (Fig. S3d,f) in young adult male

mice. While exploratory results showed that HU-308 (0.2, 1 mg/kg) reduced DOI-induced ESR (Fig. S3e). Body weights were not different between groups (Fig. S5e,f).

These observations were further supported with results from the marble burying test (MBT). In young adult male mice, HU-308 (0.2, 1, 5 mg/kg) had no effect on the distance the mice moved in the cage, latency to center or the number of buried marbles (Fig. S4a-c). There was also no difference in the time spent in the center, frequency of entries to center, or in the number of moved marbles (not shown). Similar results were obtained in young adult female mice, except that HU-308 (5 mg/kg) significantly increased the latency to center (Fig. S4d-f). Body weights were not different between groups (Fig. S2c,d).

Effect of HU-308 on basal repetitive behaviors in juvenile mice- experimental conditions

Similar results were found under different experimental conditions i.e. in which the second injection is with saline which was used as a control in the DOI vs. SR141716A model systems. Compared with the basal HTR of the control group (0.3 ± 0.2), HU-308 (0.2, 1, 5 mg/kg) significantly increased HTR to 2.0 ± 0.7, 1.3 ± 0.6 and 0.7 ± 0.2, resulting in an increase of 600%, 350% and 150% of basal HTR in juvenile mice, respectively (Fig. S9a). These results suggest that juvenile males are more sensitive to the stimulation of CB_2 receptor-induced neck/head motor-like tics than females. It was notable that the lower dose had a higher effect, suggesting that the direction of the dose-response to HU-308 depends on the presence of salts (e.g. injection of saline). Similarly, CB_2 receptor agonists were found to stimulate the release of dopamine in the presence of cations (K^+) [9].

Compared with the basal ESR of the control group (0.7 ± 0.3), HU-308 (0.2 mg/kg) significantly decreased ESR to 0.3.0 ± 0.2, resulting in inhibition of 53% of basal ESR (Fig. S9b; $P < 0.05$). Though HU-308 (5 mg/kg) significantly reduced ESR, by the end period of counting there was no difference (Fig. S9b). HU-308 alone had no effect on basal grooming

behavior (Fig. S9c). In males and females, average body weight was not different between groups (Fig. S5a,c).

In line with our results, JWH-133 and GW833972A, both selective CB_2 receptor agonists, enhance dopamine release in the presence of K^+ in slices from the dorsal striatum of rats [9]. Interestingly, this stimulatory effect has been found only in the presence of basal activity of D_2 autoreceptors [9]. Under these conditions, CB_2 receptor increases cAMP accumulation by favoring the coupling to Gαs-proteins [9]. But when D_2 autoreceptors are blocked with sulpiride (an antagonist), activation of CB_2 receptor decreases cAMP accumulation (i.e. favoring the coupling to Gαi-proteins), leading to the inhibition of dopamine release, an effect which is reversed by AM630, a CB_2 receptor selective antagonist/inverse agonist [9]. Further support for this switch in coupling has been documented in the striatum, where a similar Gαi to Gαs switch in coupling can occur between the CB_1 and D_2 receptors [10,11]. Thus, it is possible that in juveniles, in the presence of basal activity of D_2 autoreceptors (i.e. lack of dopamine), the CB_2 receptor will favor the coupling to Gαs protein.

SR141716A model system

The effects of the vehicles on SR141716A-induced repetitive behaviours were compared. SR141716A (5, 10, 20 mg/kg), dissolved in ethanol, dose dependently and significantly increased HTR and ESR behaviours but not grooming behaviour in juvenile male C57BL/6J mice (Fig. S7a-c). These results replicate another study where repetitive behaviours were simultaneously counted for 20 min following SR141716A administration (2.5, 5, 10, 20 mg/kg; ethanol vehicle) to juvenile ICR male mice [2]. However, we noted major differences between the two strains of mice. While 20 mg/kg SR141716A induced about 80 ESR in juvenile male ICR mice after 20 min, the same dose induced only about 12 ESR after 20 min (85% lower), and 23 ESR (70% lower) after 39 min in juvenile male C57BL/6J mice. Similarly, the HTR

was about 25% lower after 20 min and 50% lower after 39 min in C57BL/6J compared to ICR mice.

Compared with DMSO vehicle, the basal HTR and ESR in ethanol vehicle were higher, while grooming behaviour was lower in the control group of juvenile male C57BL/6J mice (Fig. S7a-c vs. S7d-f, respectively). SR141716A (10, 20 mg/kg) significantly increased HTR and ESR in the presence of DMSO vehicle (Fig. S7a,b vs. S7d,e, respectively). After 20 min of SR141716A administration, there was no difference in grooming behaviour in ethanol or DMSO (Fig. S7c vs. S7f, respectively). However, a small but significant effect of SR141716A on grooming behaviour was found 39 min following its injection. Grooming behaviour was significantly increased by SR141716A (10 mg/kg) in the presence of ethanol, while SR141716A (10, 20 mg/kg) significantly reduced grooming behaviour in the presence of DMSO (Fig. S7c vs. S7f, respectively). Average body weight was not different between groups (Fig. S8a-b).

As similar effect of SR141716A on HTR was found with both vehicles, but as ethanol increased basal HTR and ESR, we have used DMSO vehicle in the subsequent experiments at a selected dose of 10 mg/kg SR141716A.

Supplementary legends

Fig. S1

Sex comparison of the effect of HU-308 (5 mg/kg) on DOI-induced HTR (a, d, g, j), ESR (b, e, h, k) and grooming behaviour (c, f, i, l) in wildtype (WT) and adult $CB_2^{-/-}$ knockout mice ($CB_2^{-/-}$ mice). In a-c, the effects of HU-308 on $CB_2^{-/-}$ females. In d-f, the effects of HU-308 on $CB_2^{-/-}$ males. In g-i, the effects of HU-308 on WT females. In j-l, the effects of HU-308 on WT males. Two-way ANOVA analysis of variance followed by Bonferroni's test for multiple comparisons was performed by GraphPad Prism 8. Asterisks aside the graph are *p* value summary vs. vehicle + DOI group. Asterisks along the curve are *p* values of multiple comparisons (at a time point) of each dose vs. vehicle + DOI group. ***** $P < 0.05$; ****** $P < 0.01$; ******* $P < 0.001$ significantly different.

Fig. S2

Body weight of each adult wildtype (a) and $CB_2^{-/-}$ (b) mouse treated with DOI in the absence or presence of HU-308 pre-treatment. Body weight of each young adult male (c) and female (d) mouse treated with HU-308 before the MBT behaviour test. On the experimental day, each body weight was determined before drug injection. The average of body weights was not significantly different between the experimental groups.

Fig. S3

In young adult mice, HU-308 alone had no effect on repetitive behaviours. Effect of HU-308 (0.2, 1, 5 mg/kg) on basal and DOI-induced HTR (a, d), ESR (b, e) and grooming behaviour (c, f) in young adult male mice. In a-c, the effects of HU-308 alone. In d-f, the effects of HU-308 in the presence of DOI (1 mg/kg). Data represent mean ± SEM. *n* represent the number of animals in each group. The experiment was independently repeated a number of times according to the lowest *n* number.

Fig. S4

Effect of HU-308 (0.2, 1, 5 mg/kg) on young adult males and females in the marble burying test. HU-308 had no effect on the distance (a, d) and the number of buried marbles (c, f). HU-308 (5 mg/kg) significantly increased the latency to centre in females (e) but not in males (b). Data represent mean ± SEM. *n* represent the number of animals in each group. The experiment was independently repeated 7 times. One-way ANOVA analysis of variance followed by Bonferroni's test for multiple comparisons was performed by GraphPad Prism 8. * $P < 0.05$ significantly different.

Fig. S5

Body weight of each HU-308 pre-treated mouse in the absence (a, c, e) or presence of DOI (b, d, f). In juvenile males (a, b), juvenile females (c, d), young adult males (e, f). On the experimental day, each body weight was determined before drug injection. The average of body weights was not significantly different between the experimental groups.

Fig. S6

Sex comparison of the effect of HU-308 (0.2 mg/kg) on DOI (1 mg/kg)-induced HTR (a, d), ESR (b, e) and grooming behaviour (c, f) on juvenile male (a-c) and female (d-f) mice. In males, HU-308 (0.2 mg/kg) had no effect, while it significantly inhibited DOI-induced HTR and grooming behaviour in females. Data represent mean ± SEM. *n* represent the number of animals in each group. The experiment was independently repeated a number of times according to the lowest *n* number. Two-way ANOVA analysis of variance followed by Bonferroni's test for multiple comparisons was performed by GraphPad Prism 8. *$P < 0.05$ significantly different as indicated. Asterisks aside the graph are *p* value summary vs. vehicle + DOI group. * $P < 0.05$; ** $P < 0.01$; *** $P < 0.001$; **** $P < 0.0001$ significantly different.

Fig. S7

Effect of SR141716A on HTR (a, d), ESR (b, e) and grooming behaviour (c, f) in juvenile male mice. In a-c, the effects of SR141716A (5, 10, 20 mg/kg) in the presence of ethanol vehicle. In d-f, the effects of SR141716A (10, 20 mg/kg) in the presence of DMSO vehicle. Data represent mean ± SEM. *n* represent the number of animals in each group. The experiment was independently repeated a number of times according to the lowest *n* number. Two-way ANOVA analysis of variance followed by Bonferroni's test for multiple comparisons was performed by GraphPad Prism 8. Asterisks aside the graph are *p* value summary vs. control (respective vehicle) group. Asterisks along the curve are *p* values of multiple comparisons (at a time point) of each dose vs. vehicle group. * $P < 0.05$; ** $P < 0.01$; *** $P < 0.001$; **** $P < 0.0001$ significantly different.

Fig. S8

Body weight of each SR141716A-induced mouse in the presence of ethanol (a) or DMSO (b) vehicles. Body weight of each HU-308 pre-treated mouse in the absence (c) or presence of SR141716A (d). On the experimental day, each body weight was determined before drug injection. The average of body weights was not significantly different between the experimental groups.

Fig. S9

Effect of HU-308 (0.2, 1, 5 mg/kg) on basal HTR (a), ESR (b) and grooming behaviour (c) in juvenile male mice (experimental condition of DOI model). Data represent mean ± SEM. *n* represent the number of animals in each group. The experiment was independently repeated 6 times. Asterisks aside the graph are *p* value summary vs. control (respective vehicle) group. Asterisks along the curve are *p* values of multiple comparisons (at a time point) of each dose vs. vehicle group. * $P < 0.05$; ** $P < 0.01$; *** $P < 0.001$; **** $P < 0.0001$ significantly different.

Fig. S10

In the open field test, HU-308 alone (1, 5 mg/kg) significantly reduced locomotor activity of ambulation (a), rearing (b) but had no effect on grooming behaviour (c) in juvenile male mice. The average of body weights was not significantly different between the experimental groups (d). Data represent mean ± SEM. *n* represent the number of animals in each group. The experiment was independently repeated 5 times. Two-way ANOVA analysis of variance followed by Bonferroni's test for multiple comparisons was performed by GraphPad Prism 8. Asterisks aside the graph are *p* value summary vs. control group (vehicle + vehicle). Asterisks

along the curve are *p* values of multiple comparisons (at a time point) of each dose vs. the control group. * $P < 0.05$; ** $P < 0.01$; *** $P < 0.001$; **** $P < 0.0001$ significantly different.

Supplementary reference

1. Gorberg V, McCaffery P, Anavi-Goffer S. Different responses of repetitive behaviours in juvenile and young adult mice to Delta(9) -tetrahydrocannabinol and cannabidiol may affect decision making for Tourette syndrome. *Br J Pharmacol*, 178(3), 614-625 (2021).
2. Darmani NA, Pandya DK. Involvement of other neurotransmitters in behaviors induced by the cannabinoid CB1 receptor antagonist SR 141716A in naive mice. *J Neural Transm (Vienna)*, 107(8-9), 931-945 (2000).
3. Darmani NA. Cannabinoids of diverse structure inhibit two DOI-induced 5-HT(2A) receptor-mediated behaviors in mice. *Pharmacol Biochem Behav*, 68(2), 311-317 (2001).
4. Hanus L, Breuer A, Tchilibon S *et al.* HU-308: a specific agonist for CB(2), a peripheral cannabinoid receptor. *Proc Natl Acad Sci U S A*, 96(25), 14228-14233 (1999).
5. Deiana S, Watanabe A, Yamasaki Y *et al.* Plasma and brain pharmacokinetic profile of cannabidiol (CBD), cannabidivarine (CBDV), Delta(9)-tetrahydrocannabivarin (THCV) and cannabigerol (CBG) in rats and mice following oral and intraperitoneal administration and CBD action on obsessive-compulsive behaviour. *Psychopharmacology (Berl)*, 219(3), 859-873 (2012).
6. Kinsey SG, O'Neal ST, Long JZ, Cravatt BF, Lichtman AH. Inhibition of endocannabinoid catabolic enzymes elicits anxiolytic-like effects in the marble burying assay. *Pharmacol Biochem Behav*, 98(1), 21-27 (2011).
7. Coutts AA, Anavi-Goffer S, Ross RA *et al.* Agonist-induced internalization and trafficking of cannabinoid CB1 receptors in hippocampal neurons. *J Neurosci*, 21(7), 2425-2433 (2001).

8. Livak KJ, Schmittgen TD. Analysis of relative gene expression data using real-time quantitative PCR and the 2(-Delta Delta C(T)) Method. *Methods (San Diego, Calif.)*, 25(4), 402-408 (2001).

9. Lopez-Ramirez G, Sanchez-Zavaleta R, Avalos-Fuentes A *et al.* D2 autoreceptor switches CB2 receptor effects on [(3) H]-dopamine release in the striatum. *Synapse*, e22139 (2019).

10. Glass M, Felder CC. Concurrent stimulation of cannabinoid CB1 and dopamine D2 receptors augments cAMP accumulation in striatal neurons: evidence for a Gs linkage to the CB1 receptor. *J Neurosci*, 17(14), 5327-5333 (1997).

11. Kearn CS, Blake-Palmer K, Daniel E, Mackie K, Glass M. Concurrent stimulation of cannabinoid CB1 and dopamine D2 receptors enhances heterodimer formation: a mechanism for receptor cross-talk? *Mol Pharmacol*, 67(5), 1697-1704 (2005).

Figure S1

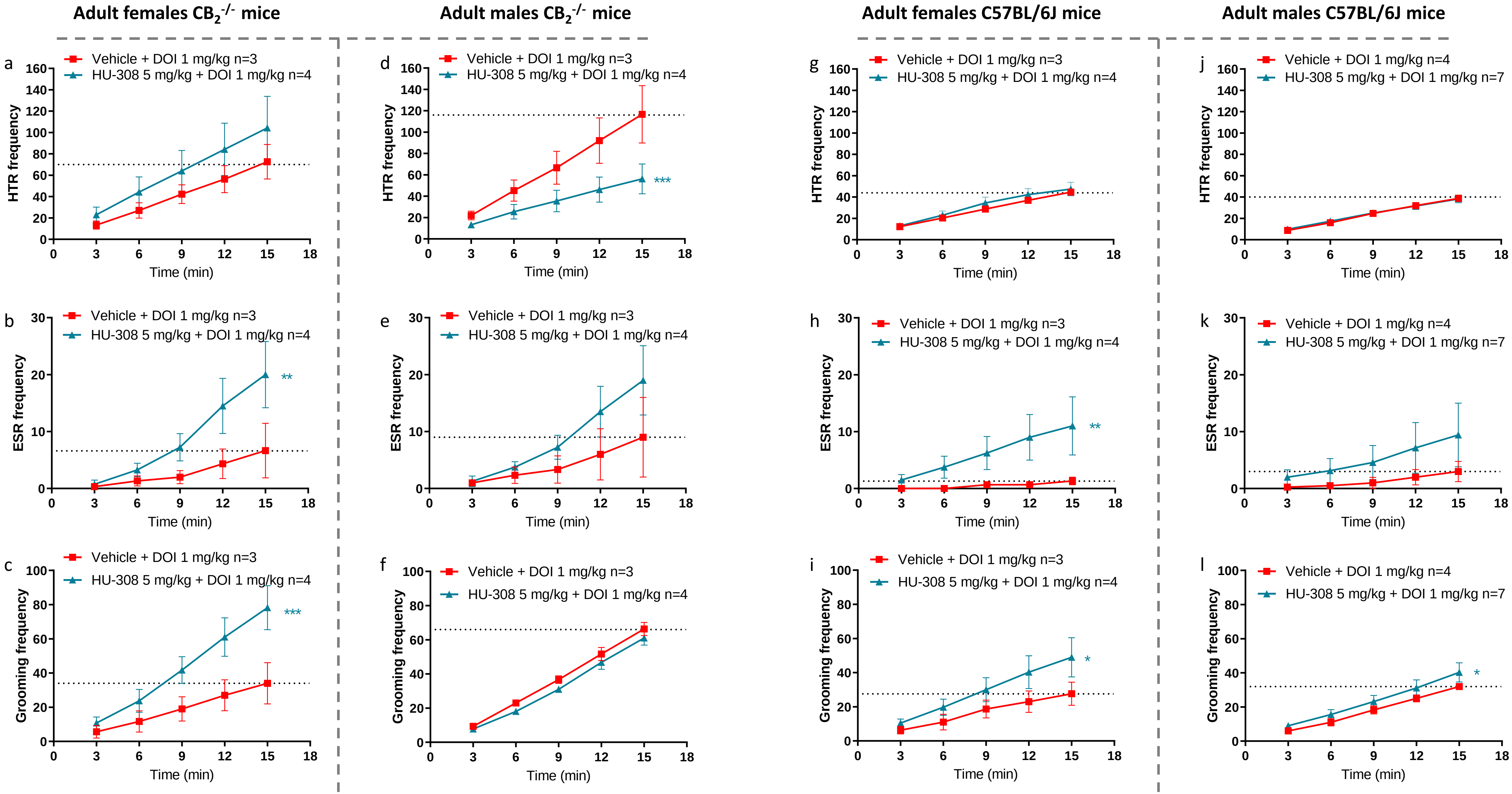

Adult females CB2-/- mice
Adult males CB2-/- mice
Adult females C57BL/6J mice
Adult males C57BL/6J mice
a
b
c
d
e
f
g
h
i
j
k
l
Vehicle + DOI 1 mg/kg n=3
HU-308 5 mg/kg + DOI 1 mg/kg n=4
Vehicle + DOI 1 mg/kg n=4
HU-308 5 mg/kg + DOI 1 mg/kg n=7
HTR frequency
ESR frequency
Grooming frequency
Time (min)
**
***
*

Figure S2

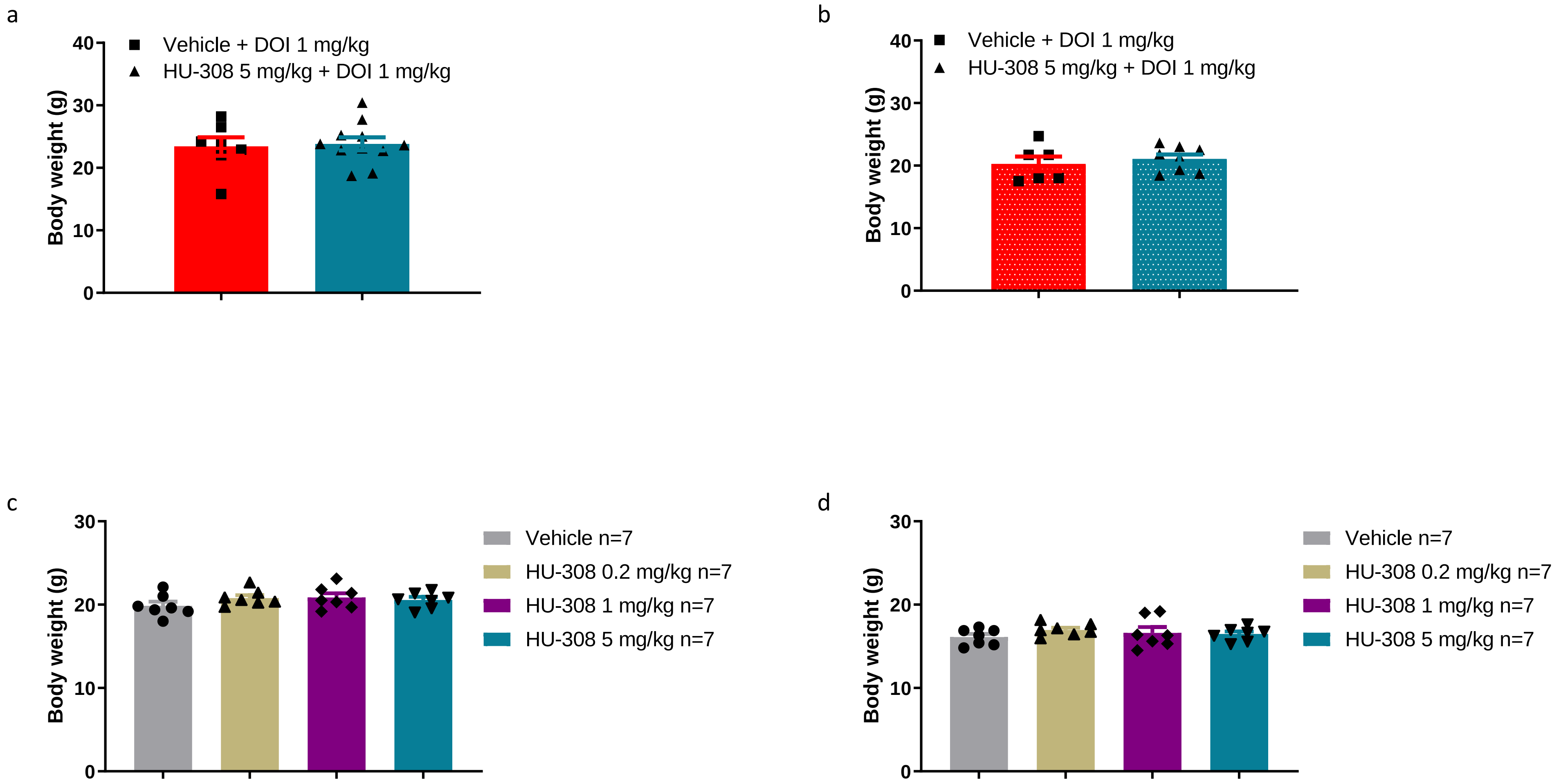
a
Vehicle + DOI 1 mg/kg
HU-308 5 mg/kg + DOI 1 mg/kg
Body weight (g)
40
30
20
10
0
b
Vehicle + DOI 1 mg/kg
HU-308 5 mg/kg + DOI 1 mg/kg
Body weight (g)
40
30
20
10
0
c
Vehicle n=7
HU-308 0.2 mg/kg n=7
HU-308 1 mg/kg n=7
HU-308 5 mg/kg n=7
Body weight (g)
30
20
10
0
d
Vehicle n=7
HU-308 0.2 mg/kg n=7
HU-308 1 mg/kg n=7
HU-308 5 mg/kg n=7
Body weight (g)
30
20
10
0

Figure S3

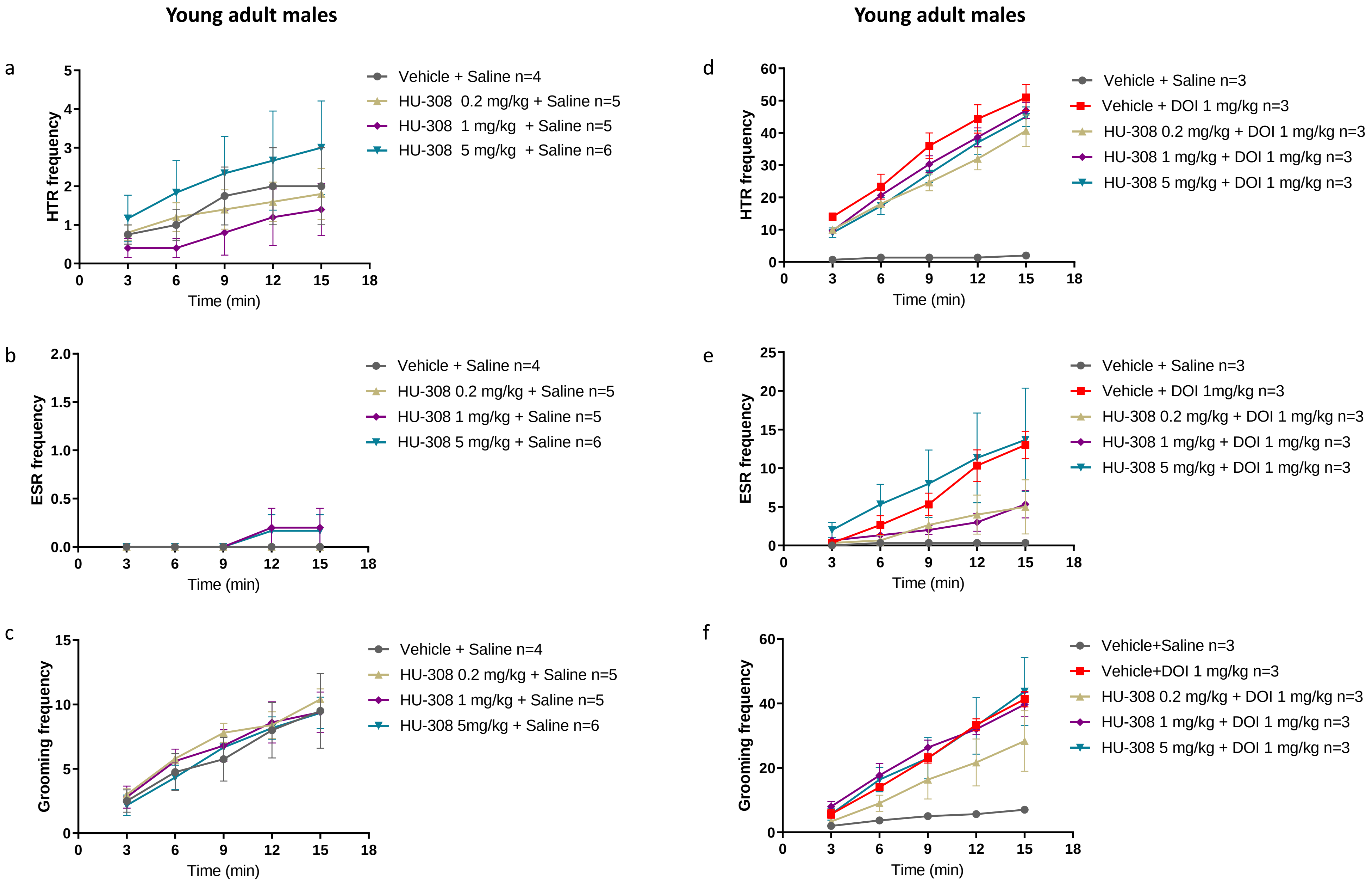

Young adult males
Young adult males
a
HTR frequency
Time (min)
Vehicle + Saline n=4
HU-308 0.2 mg/kg + Saline n=5
HU-308 1 mg/kg + Saline n=5
HU-308 5 mg/kg + Saline n=6
b
ESR frequency
Time (min)
Vehicle + Saline n=4
HU-308 0.2 mg/kg + Saline n=5
HU-308 1 mg/kg + Saline n=5
HU-308 5 mg/kg + Saline n=6
c
Grooming frequency
Time (min)
Vehicle + Saline n=4
HU-308 0.2 mg/kg + Saline n=5
HU-308 1 mg/kg + Saline n=5
HU-308 5mg/kg + Saline n=6
d
HTR frequency
Time (min)
Vehicle + Saline n=3
Vehicle + DOI 1 mg/kg n=3
HU-308 0.2 mg/kg + DOI 1 mg/kg n=3
HU-308 1 mg/kg + DOI 1 mg/kg n=3
HU-308 5 mg/kg + DOI 1 mg/kg n=3
e
ESR frequency
Time (min)
Vehicle + Saline n=3
Vehicle + DOI 1mg/kg n=3
HU-308 0.2 mg/kg + DOI 1 mg/kg n=3
HU-308 1 mg/kg + DOI 1 mg/kg n=3
HU-308 5 mg/kg + DOI 1 mg/kg n=3
f
Grooming frequency
Time (min)
Vehicle+Saline n=3
Vehicle+DOI 1 mg/kg n=3
HU-308 0.2 mg/kg + DOI 1 mg/kg n=3
HU-308 1 mg/kg + DOI 1 mg/kg n=3
HU-308 5 mg/kg + DOI 1 mg/kg n=3

Figure S4

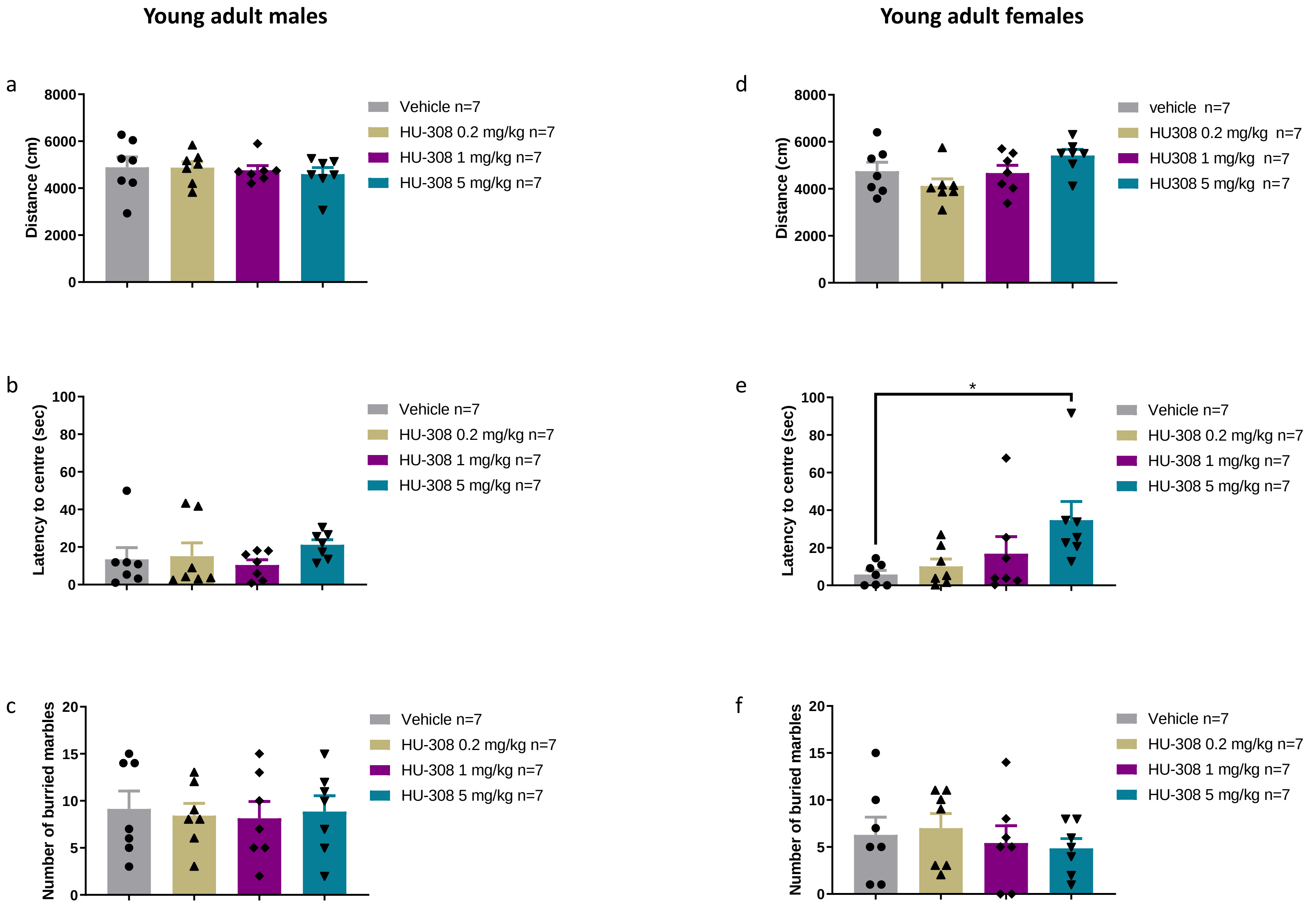
Young adult males
Young adult females
a
Distance (cm)
8000
6000
4000
2000
0
Vehicle n=7
HU-308 0.2 mg/kg n=7
HU-308 1 mg/kg n=7
HU-308 5 mg/kg n=7
d
vehicle n=7
HU308 0.2 mg/kg n=7
HU308 1 mg/kg n=7
HU308 5 mg/kg n=7
b
Latency to centre (sec)
100
80
60
40
20
0
e
*
c
Number of burried marbles
20
15
10
5
0
f
Number of buried marbles

Figure S5

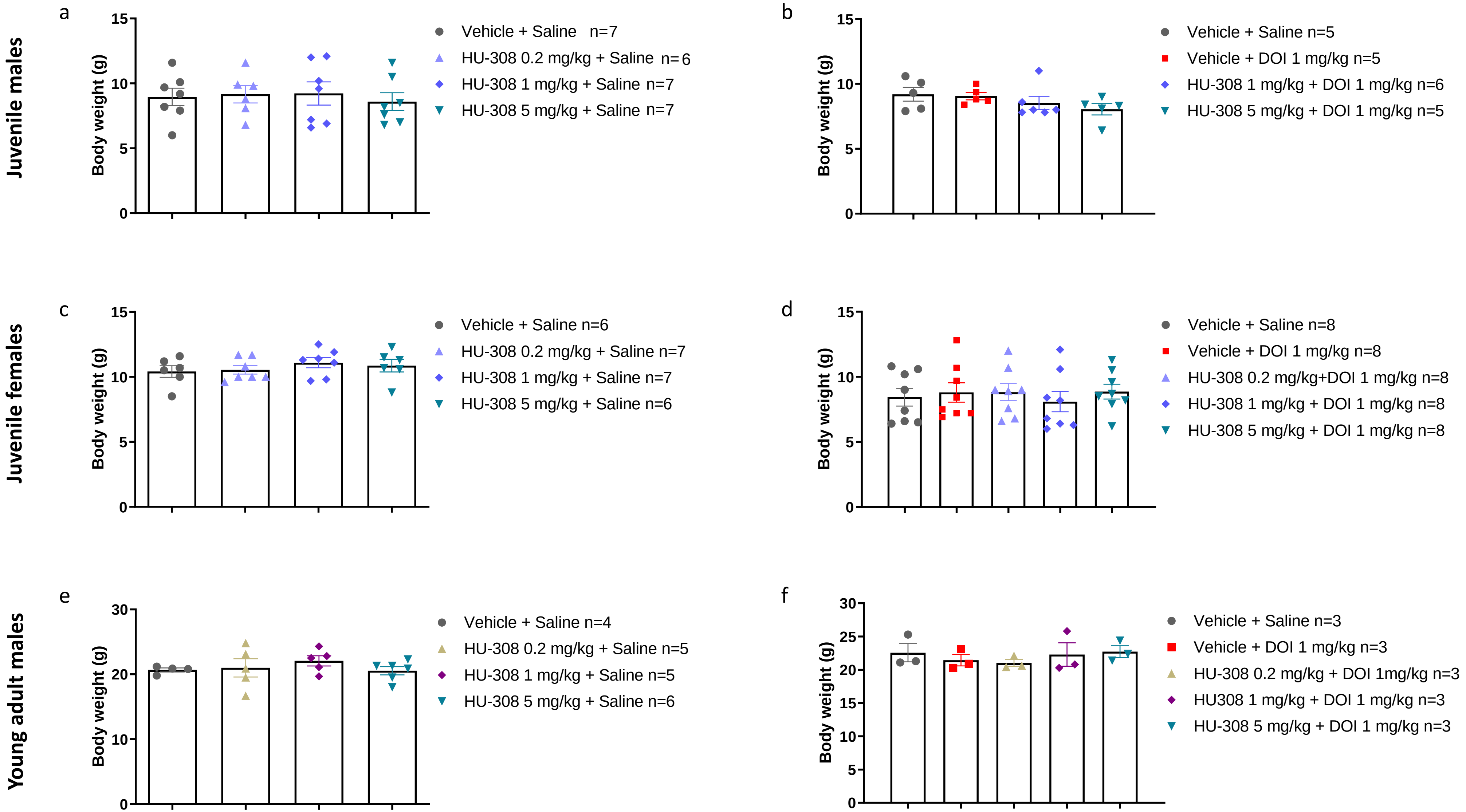
Juvenile males
a
Body weight (g)
15
10
5
0
Vehicle + Saline n=7
HU-308 0.2 mg/kg + Saline n= 6
HU-308 1 mg/kg + Saline n=7
HU-308 5 mg/kg + Saline n=7
b
Body weight (g)
Vehicle + Saline n=5
Vehicle + DOI 1 mg/kg n=5
HU-308 1 mg/kg + DOI 1 mg/kg n=6
HU-308 5 mg/kg + DOI 1 mg/kg n=5
Juvenile females
c
Body weight (g)
Vehicle + Saline n=6
HU-308 0.2 mg/kg + Saline n=7
HU-308 1 mg/kg + Saline n=7
HU-308 5 mg/kg + Saline n=6
d
Body weight (g)
Vehicle + Saline n=8
Vehicle + DOI 1 mg/kg n=8
HU-308 0.2 mg/kg+DOI 1 mg/kg n=8
HU-308 1 mg/kg + DOI 1 mg/kg n=8
HU-308 5 mg/kg + DOI 1 mg/kg n=8
Young adult males
e
Body weight (g)
30
20
10
0
Vehicle + Saline n=4
HU-308 0.2 mg/kg + Saline n=5
HU-308 1 mg/kg + Saline n=5
HU-308 5 mg/kg + Saline n=6
f
Body weight (g)
30
25
20
15
10
5
0
Vehicle + Saline n=3
Vehicle + DOI 1 mg/kg n=3
HU-308 0.2 mg/kg + DOI 1mg/kg n=3
HU308 1 mg/kg + DOI 1 mg/kg n=3
HU-308 5 mg/kg + DOI 1 mg/kg n=3

Figure S6

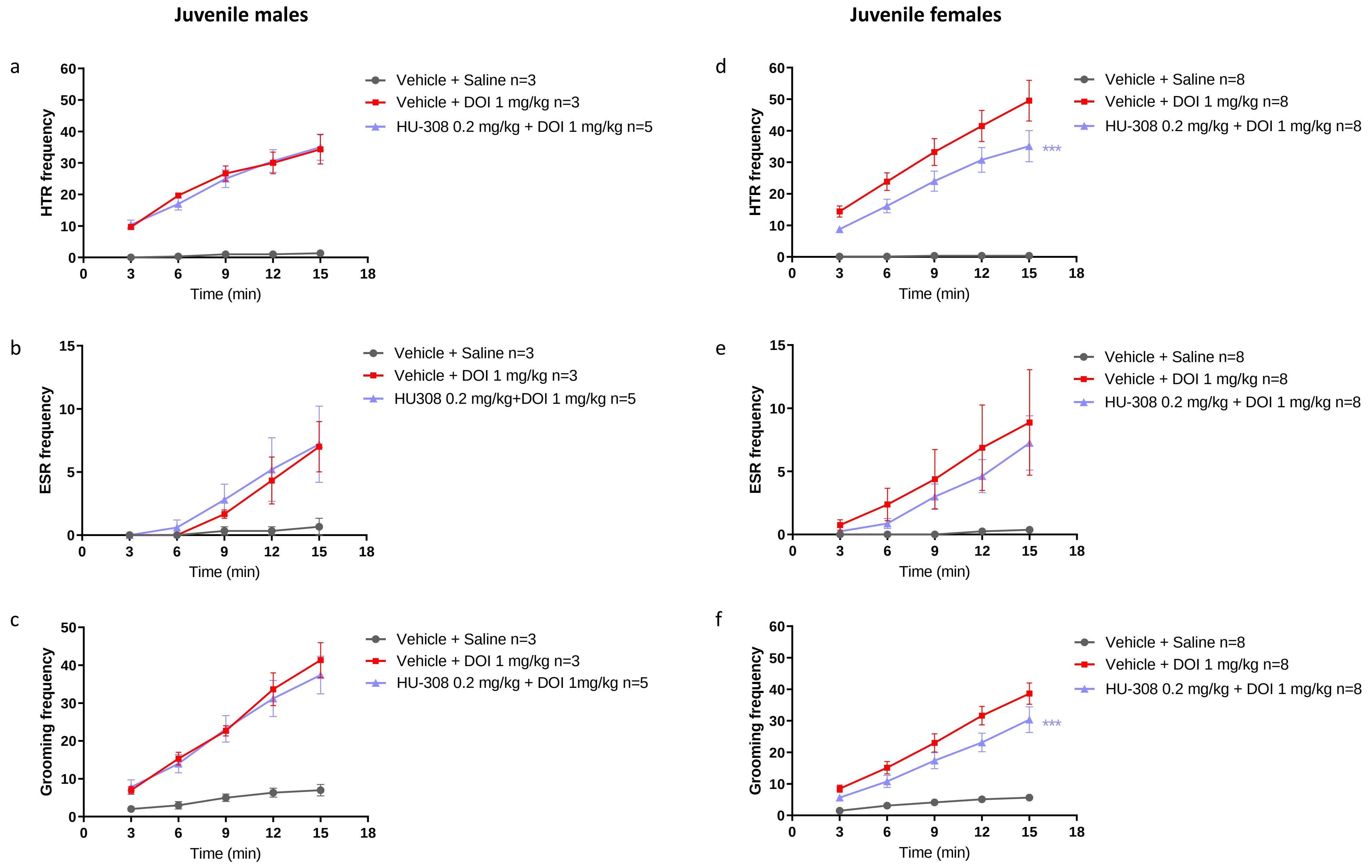

Juvenile males
Juvenile females
a
HTR frequency
Time (min)
Vehicle + Saline n=3
Vehicle + DOI 1 mg/kg n=3
HU-308 0.2 mg/kg + DOI 1 mg/kg n=5
b
ESR frequency
Time (min)
Vehicle + Saline n=3
Vehicle + DOI 1 mg/kg n=3
HU308 0.2 mg/kg+DOI 1 mg/kg n=5
c
Grooming frequency
Time (min)
Vehicle + Saline n=3
Vehicle + DOI 1 mg/kg n=3
HU-308 0.2 mg/kg + DOI 1mg/kg n=5
d
HTR frequency
Time (min)
***
Vehicle + Saline n=8
Vehicle + DOI 1 mg/kg n=8
HU-308 0.2 mg/kg + DOI 1 mg/kg n=8
e
ESR frequency
Time (min)
Vehicle + Saline n=8
Vehicle + DOI 1 mg/kg n=8
HU-308 0.2 mg/kg + DOI 1 mg/kg n=8
f
Grooming frequency
Time (min)
***
Vehicle + Saline n=8
Vehicle + DOI 1 mg/kg n=8
HU-308 0.2 mg/kg + DOI 1 mg/kg n=8

Figure S7

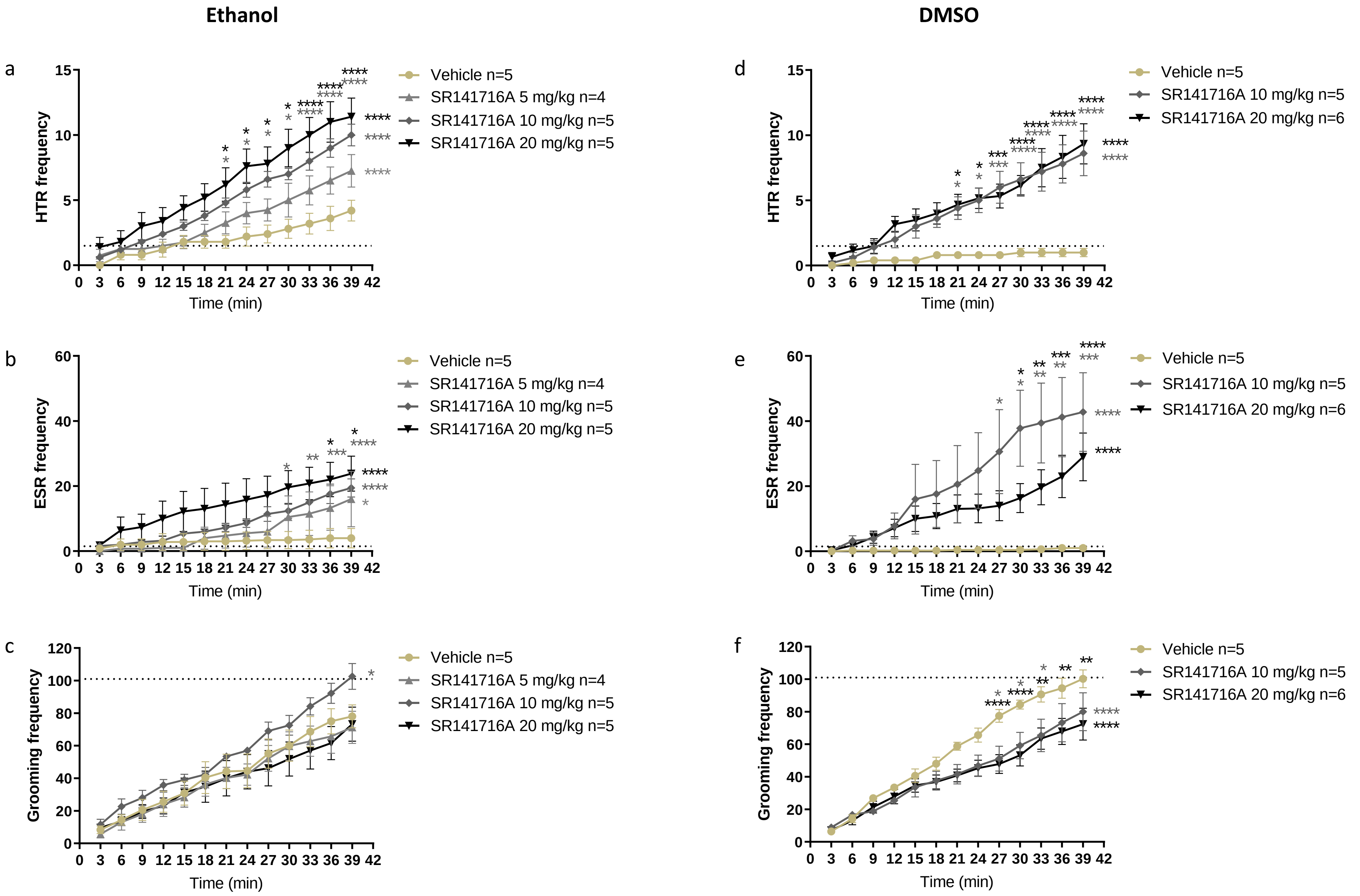
Ethanol
DMSO
a
HTR frequency
Time (min)
Vehicle n=5
SR141716A 5 mg/kg n=4
SR141716A 10 mg/kg n=5
SR141716A 20 mg/kg n=5
b
ESR frequency
Time (min)
Vehicle n=5
SR141716A 5 mg/kg n=4
SR141716A 10 mg/kg n=5
SR141716A 20 mg/kg n=5
c
Grooming frequency
Time (min)
Vehicle n=5
SR141716A 5 mg/kg n=4
SR141716A 10 mg/kg n=5
SR141716A 20 mg/kg n=5
d
HTR frequency
Time (min)
Vehicle n=5
SR141716A 10 mg/kg n=5
SR141716A 20 mg/kg n=6
e
ESR frequency
Time (min)
Vehicle n=5
SR141716A 10 mg/kg n=5
SR141716A 20 mg/kg n=6
f
Grooming frequency
Time (min)
Vehicle n=5
SR141716A 10 mg/kg n=5
SR141716A 20 mg/kg n=6

Figure S8

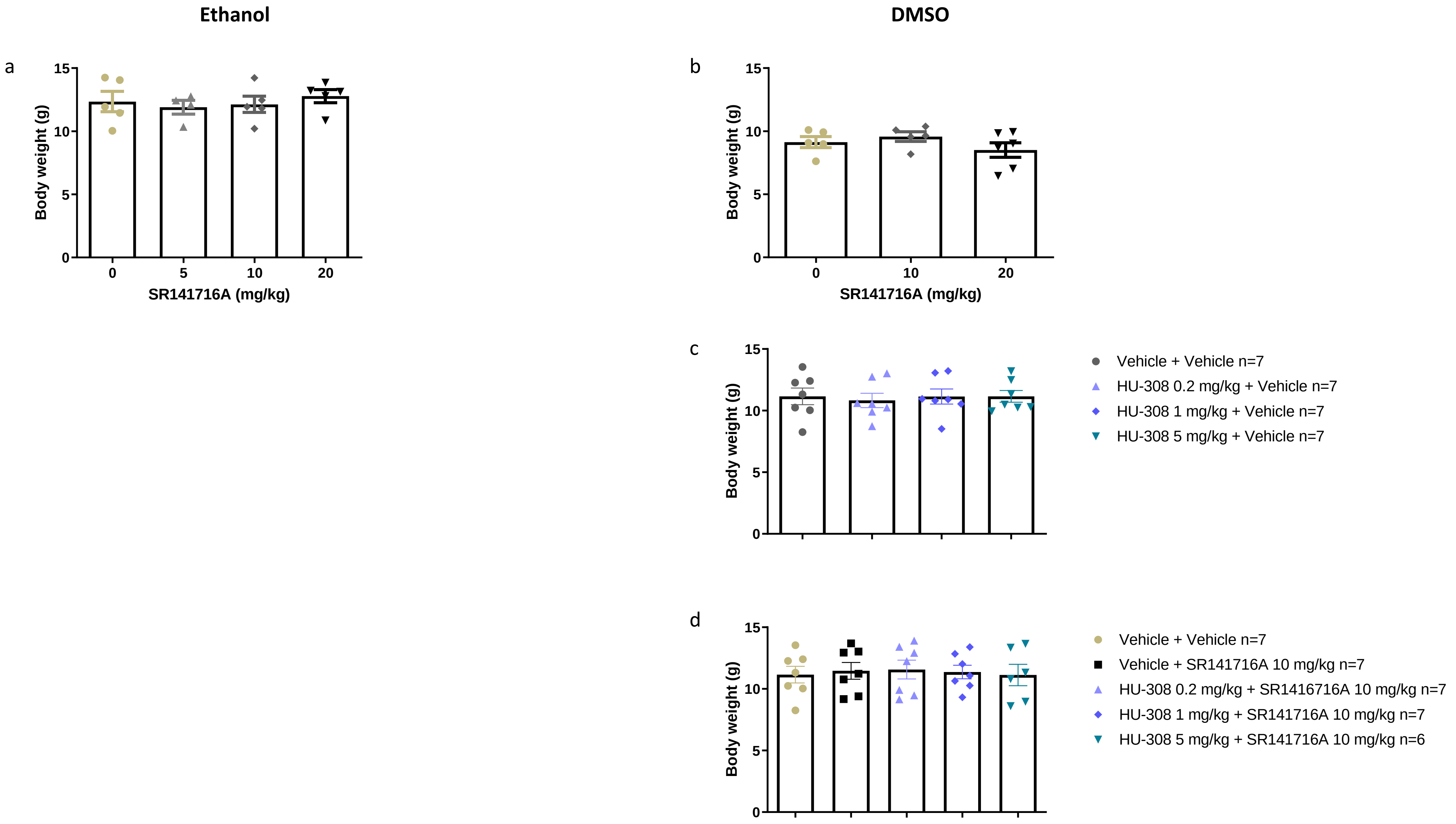

Ethanol
DMSO
a
b
c
d
Body weight (g)
SR141716A (mg/kg)
0
5
10
15
20
Vehicle + Vehicle n=7
HU-308 0.2 mg/kg + Vehicle n=7
HU-308 1 mg/kg + Vehicle n=7
HU-308 5 mg/kg + Vehicle n=7
Vehicle + Vehicle n=7
Vehicle + SR141716A 10 mg/kg n=7
HU-308 0.2 mg/kg + SR1416716A 10 mg/kg n=7
HU-308 1 mg/kg + SR141716A 10 mg/kg n=7
HU-308 5 mg/kg + SR141716A 10 mg/kg n=6

Figure S9

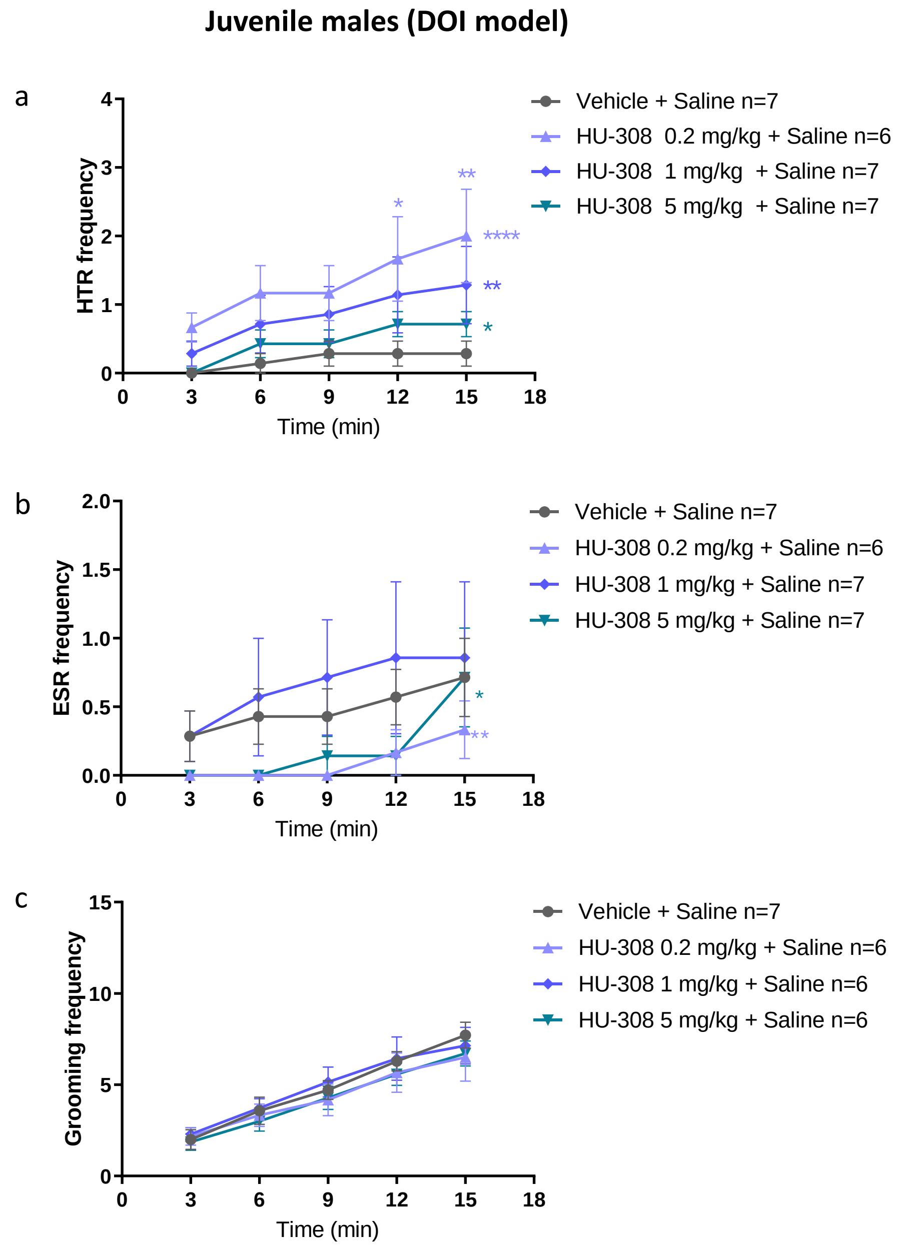
Juvenile males (DOI model)
a
HTR frequency
Time (min)
Vehicle + Saline n=7
HU-308 0.2 mg/kg + Saline n=6
HU-308 1 mg/kg + Saline n=7
HU-308 5 mg/kg + Saline n=7
b
ESR frequency
Time (min)
Vehicle + Saline n=7
HU-308 0.2 mg/kg + Saline n=6
HU-308 1 mg/kg + Saline n=7
HU-308 5 mg/kg + Saline n=7
c
Grooming frequency
Time (min)
Vehicle + Saline n=7
HU-308 0.2 mg/kg + Saline n=6
HU-308 1 mg/kg + Saline n=6
HU-308 5 mg/kg + Saline n=6

Figure S10

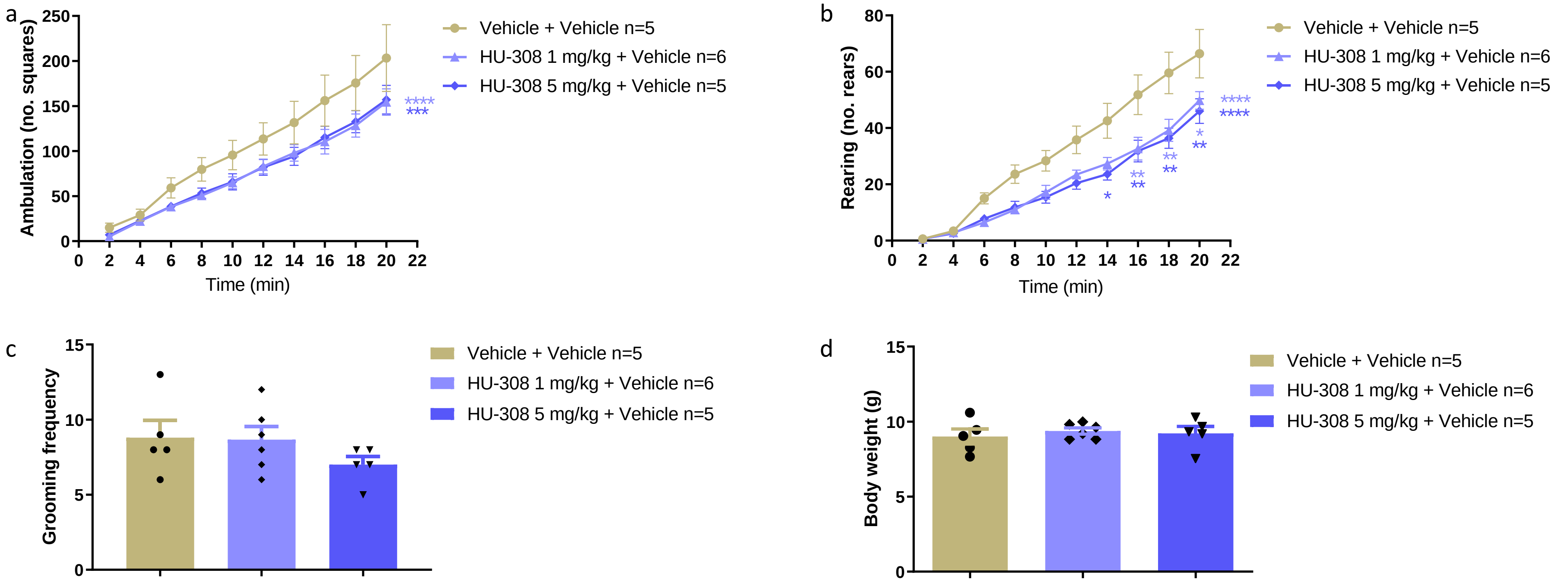
a
Ambulation (no. squares)
250
200
150
100
50
0
0 2 4 6 8 10 12 14 16 18 20 22
Time (min)
****
***
Vehicle + Vehicle n=5
HU-308 1 mg/kg + Vehicle n=6
HU-308 5 mg/kg + Vehicle n=5
b
Rearing (no. rears)
80
60
40
20
0
0 2 4 6 8 10 12 14 16 18 20 22
Time (min)
****
****
Vehicle + Vehicle n=5
HU-308 1 mg/kg + Vehicle n=6
HU-308 5 mg/kg + Vehicle n=5
c
Grooming frequency
15
10
5
0
Vehicle + Vehicle n=5
HU-308 1 mg/kg + Vehicle n=6
HU-308 5 mg/kg + Vehicle n=5
d
Body weight (g)
15
10
5
0
Vehicle + Vehicle n=5
HU-308 1 mg/kg + Vehicle n=6
HU-308 5 mg/kg + Vehicle n=5
